# Aberrant splicing in Huntington’s disease accompanies disrupted TDP-43 activity and altered m6A RNA modification

**DOI:** 10.1038/s41593-024-01850-w

**Published:** 2025-01-06

**Authors:** Thai B. Nguyen, Ricardo Miramontes, Carlos Chillon-Marinas, Roy Maimon, Sonia Vazquez-Sanchez, Alice L. Lau, Nicolette R. McClure, Zhuoxing Wu, Keona Q. Wang, Whitney E. England, Monika Singha, Jennifer T. Stocksdale, Marie Heath, Ki-Hong Jang, Sunhee Jung, Karen Ling, Paymann Jafar-nejad, Jharrayne I. McKnight, Leanne N. Ho, Osama Al Dalahmah, Richard L. M. Faull, Joan S. Steffan, Jack C. Reidling, Cholsoon Jang, Gina Lee, Don W. Cleveland, Clotilde Lagier-Tourenne, Robert C. Spitale, Leslie M. Thompson

**Affiliations:** 1 Department of Neurobiology & Behavior, University of California, Irvine, Irvine, CA USA; 2 UCI MIND, University of California, Irvine, Irvine, CA USA; 3 Department of Cellular and Molecular Medicine, University of California, San Diego, La Jolla, CA USA; 4 Department of Psychiatry & Human Behavior, University of California, Irvine, Irvine, CA USA; 5 Department of Biological Chemistry, Chao Family Comprehensive Cancer Center, School of Medicine, University of California, Irvine, Irvine, CA USA; 6 Department of Pharmaceutical Sciences, University of California, Irvine, Irvine, CA USA; 7 Department of Microbiology and Molecular Genetics, Chao Family Comprehensive Cancer Center, School of Medicine, University of California, Irvine, Irvine, CA USA; 8 Ionis Pharmaceuticals, Inc., Carlsbad, CA USA; 9 Department of Pathology and Cell Biology, Columbia University, New York, NY USA; 10 Department of Anatomy and Medical Imaging, Faculty of Medical and Health Science, University of Auckland, Auckland, New Zealand; 11 Centre for Brain Research, Faculty of Medical and Health Science, University of Auckland, Auckland, New Zealand; 12 Department of Neurology, Sean M. Healey & AMG Center for ALS, Massachusetts General Hospital, Harvard Medical School, Boston, MA USA; 13 Broad Institute of Harvard University and MIT, Cambridge, MA USA; 14 Department of Chemistry, University of California, Irvine, Irvine, CA USA; 15 Sue and Bill Gross Stem Cell Center, University of California, Irvine, Irvine, CA USA

**Keywords:** Molecular neuroscience, Diseases

## Abstract

Huntington’s disease (HD) is caused by a CAG repeat expansion in the *HTT* gene, leading to altered gene expression. However, the mechanisms leading to disrupted RNA processing in HD remain unclear. Here we identify TDP-43 and the N6-methyladenosine (m6A) writer protein METTL3 to be upstream regulators of exon skipping in multiple HD systems. Disrupted nuclear localization of TDP-43 and cytoplasmic accumulation of phosphorylated TDP-43 occurs in HD mouse and human brains, with TDP-43 also co-localizing with HTT nuclear aggregate-like bodies distinct from mutant HTT inclusions. The binding of TDP-43 onto RNAs encoding HD-associated differentially expressed and aberrantly spliced genes is decreased. Finally, m6A RNA modification is reduced on RNAs abnormally expressed in the striatum of HD R6/2 mouse brain, including at clustered sites adjacent to TDP-43 binding sites. Our evidence supports TDP-43 loss of function coupled with altered m6A modification as a mechanism underlying alternative splicing in HD.

## Abstract (web summary)

Nguyen et al. identify TDP-43 and METTL3 as key regulators of disrupted RNA splicing in Huntington’s disease, offering insight into how TDP-43 mislocalization and aberrant m6A RNA modification and localization relate to disease pathogenesis.

## Main

Huntington’s disease (HD) is an autosomal dominant neurodegenerative disorder that manifests with motor, cognitive and psychiatric symptoms^1–3^. HD is caused by a CAG repeat expansion mutation in exon 1 of the *HTT* (huntingtin) gene, which subsequently encodes an expanded polyglutamine repeat within the huntingtin (HTT) protein (mHTT)^1^. The most overt pathological phenotypes include loss of striatal neurons, primarily the GABAergic medium spiny neurons (MSNs), and cortical atrophy^4^. Somatic repeat instability occurs in the striatum^5–9^, and the expansion is linked to both gain of pathogenic functions and disruption of normal HTT function^10^. The CAG expansion in HD also promotes the formation of an aberrant and toxic splice product through incomplete splicing of exon 1, *mHTT*_ex1_ (ref. ^11^), and accumulation of repeat-associated non-ATG (RAN) translation proteins^12^. To date, no disease-modifying treatment is available.

HTT protein interactors and how those interactions change in the context of mHTT expression have been documented in yeast, mammalian cells, mouse brains and human brains^13^. One class of HTT protein interactors is represented by RNA-binding proteins (RBPs), which are responsible for all aspects of RNA processing^14,15^. Of the published work on HTT and RBPs, most (for example FUS^16^, G3BP1 (refs. ^17,18^), CAPRIN1 (ref. ^17^) and TDP-43 (refs. ^18–20^)) have been linked to the regulation of the oxidative stress response and aggregation. Although abnormal interactions between HTT and RBPs were reported, suggesting disruption of RNA processing in HD, the mechanisms by which mHTT leads to alterations of RNA expression and splicing—a hallmark of HD and other neuropathological disorders—remain undetermined.

TDP-43 (*TARDBP*) is a DNA/RNA-binding protein critical for splicing regulation that is mutated and/or mislocalized in amyotrophic lateral sclerosis (ALS) and frontotemporal lobar degeneration (FTLD)^21–23^. Indeed, a striking nuclear-to-cytoplasmic translocation of TDP-43 is the pathological hallmark in 97% of sporadic ALS (sALS) cases and in up to 50% of patients with sporadic and familial FTLD^21,24–26^. TDP-43 mislocalization is also found in up to 50% of Alzheimer’s disease (AD) cases^25,27^, in a subset of patients with Parkinson’s disease^28,29^ and in the brains of patients with HD^19,30^. In HD, TDP-43 has been reported to be incorporated into HTT EM48-positive intracellular inclusions^19^; these inclusions were found to promote somatic CAG repeat expansion in HD mice^18^. Loss of nuclear TDP-43 results in abnormal splicing events^23,25,31,32^, including the aberrant inclusion of cryptic exons (CEs)^33–37^. It is unknown whether HD-associated expression and splicing alterations are related to TDP-43 disruption in HD.

The most abundant chemical modification of mRNA—N6-methyladenosine (m6A)—occurs on the adenosine base at the N6 position, altering localization, splicing, stability and translational control^38–48^. Adenosine is methylated to yield m6A by writer proteins (METTL3, METTL14, WTAP and RBM15) and reversed by eraser proteins (FTO and ALKBH5). m6A attracts and repels RBPs to modulate the binding and regulation of the target RNA^49^ and regulates neural development^45,50^. It was recently shown that postmortem tissues from patients with ALS display widespread RNA hypermethylation and that TDP-43 preferentially binds m6A-modified RNAs^51^. Additionally, altered m6A patterns have been reported in the hippocampus of the Q111 knock-in mouse model of HD with effects on behavior^52^.

In the present study, we used RNA sequencing (RNA-seq) analysis of brain tissue from the HD R6/2 transgenic model^53^ that expresses human mHTTex1 to identify alterations in RNA processing, including RNA splicing. Splicing-specific changes were also identified in HD Q150 (refs. ^54,55^) and Q175 (ref. ^56^) knock-in mouse models. Novel processing alterations were confirmed by long-read RNA-seq. We identified primary sequence motifs—RNA binding sites associated with mHTT-dependent changes in alternative splicing (AS) using primary sequence models—that implicated two HTT-interacting RBPs: TDP-43 and methyltransferase 3 (METTL3). Using molecular and neuropathological measures and TDP-43 enhanced crosslinking and immunoprecipitation sequencing (eCLIP-seq) and m6A eCLIP-seq, we determined that mHTT disrupts TDP-43 and METTL3 function in post-transcriptional processing of their RNA targets in HD. We further show a nuclear aggregate-like structure in the brains of patients with HD that contain TDP-43 and HTT. This study provides evidence for functional disruption of TDP-43 in HD and an association with abnormal m6A RNA modification in HD. This work also suggests that TDP-43 dysregulation may be an important component of pathogenesis in a broader group of diseases than previously thought.

## Results

### RNA processing is dysregulated in HD R6/2 mouse brains

HTT and mHTT protein–protein interactors have been reported from human, mouse, cell and yeast and were curated into a list of 4,182 predicted protein interactors (https://www.hdinhd.org/)^13^. We found that the top enriched Gene Ontology (GO) function term was RNA binding, with 788 interactors (18.8%) being known or predicted RBPs (42.9%; 788/1,836)^57^ (Supplementary Fig. 1a; RBP interactors of HTT in Supplementary Data 1). RNA-seq was performed to analyze splicing alterations in 10 3-month-old (3mos) (five males, five females; R6/2 versus non-transgenic (NT)) symptomatic HD R6/2 transgenic^53^ and NT control mice, because this model’s transcriptional signature recapitulates many human HD transcriptional changes^58^. Principal component analysis (PCA) showed the separation of genotypes across PC1 and sex across PC2 (Supplementary Fig. 1b). Differential gene expression (DGE) analysis accounting for sex differences resulted in 1,309 downregulated genes and 648 upregulated genes in the striatum (adjusted *P* < 0.05 and log_2_foldchange (FC) > 1) and 1,253 downregulated genes and 462 upregulated genes in the cortex (significant differentially expressed genes (DEGs) with adjusted *P* < 0.05 in Supplementary Data 2). A 65.8% overlap between the striatal and cortical DEGs was observed, with 95.4% changing in the same direction (Supplementary Fig. 1c). An HD striatal transcriptome signature of 266 disease-associated expression changes was previously identified from multiple mouse models of HD (HD266ssg)^59^. Our striatal RNA-seq results showed dysregulation of all 266, with 265 in the anticipated direction. The cortical transcriptome signature generally mirrored the striatal 266 genes (Supplementary Fig. 1d; full 266 gene list in Supplementary Data 3). Strengthening our hypothesis that HTT’s interactions with RBPs may drive HD R6/2 transcriptional signatures in the cortex and striatum, we observed upregulated DEGs in both regions to be enriched for GO terms relating to RNA metabolism, RNA processing and RNA splicing (Supplementary Fig. 1e,f).

### Increased exon exclusion in HD mouse models and human brain tissues

**Fig. 1:**
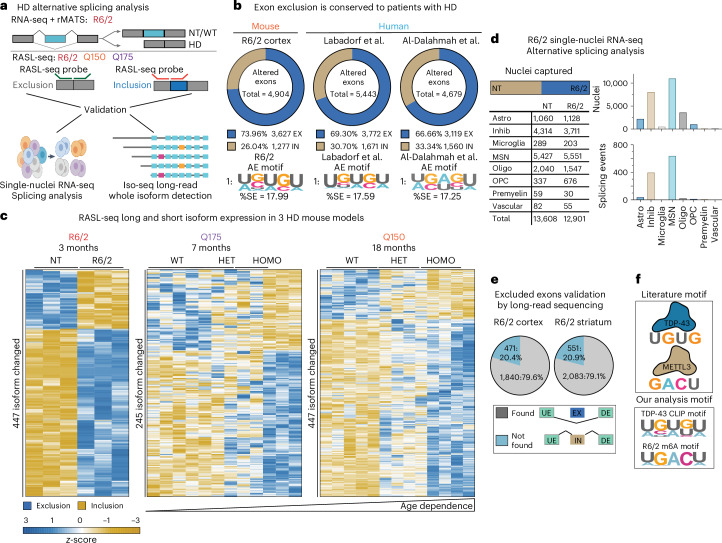
Aberrant AS in HD mice. **a**, Schematics of detection of alternatively spliced exons in HD mouse models with RASL-seq^98^ and rMATS^60^. **b**, Altered exon (AE) events annotated by rMATS: inclusion (IN) or exclusion (EX) detected in 3mos R6/2 cortex (left) and using two published HD RNA-seq datasets from Labadorf et al.^78^ and Al-Dalahmah et al.^63^ (prefrontal cortex BA9, grade III/IV, 20 HD, 49 controls; anterior cingulate cortex, grade III/IV, six HD, six controls). Top-ranked HOMER enriched motif for each dataset is shown at the bottom. Splicing events were filtered for FDR < 0.05. **c**, Heatmap showing the detection of the short isoform (excluded exon) and long isoform (included exon) from RASL-seq in cortex from the R6/2, Q175 and Q150 HD mouse models. Gradient scale represents z-scores of normalized gene counts. NT, non-transgenic control for the R6/2; WT, wild type; HET, heterozygous (Q7/Q175, Q7/Q150); HOMO, homozygous (Q175/Q175, Q150/Q150). **d**, Left: single-nuclei sequencing validation; cell type classification of total nuclei sequenced from the striatum of 3mos R6/2. Right: bar graphs show nuclei counts from single-nuclei sequencing and number of significant (*P* < 0.05) splicing events detected in cell types from DESJ-detection^66^. From top to bottom: Astro, astrocytes; Inhib, inhibitory neurons; microglia; Oligo, oligodendrocytes; OPC, oligodendrocyte progenitor cells; premyelin cells; and vascular cells. **e**, Pie chart showing the detection of significant excluded exons in PacBio Iso-seq long-read sequencing. UE, upstream exon; DE, downstream exon; IN, included exon; EX, excluded exon. **f**, Canonical binding motif for TDP-43 (UG rich) and METTL3 (DRACH).

Next, we set out to understand AS changes in the HD mouse model (Fig. 1a). Applying the AS tool (RNA-seq multivariate analysis of transcript splicing (rMATS)^60^) on R6/2 and age-matched NT RNA-seq data from 3mos animals, 9,033 RNAs (3,620 genes) with significant splicing events (false discovery rate (FDR) < 0.05) were identified in the cortex and 10,074 RNAs (3,854 genes) in the striatum (Supplementary Fig. 1g). These are classified by rMATS into five canonical splicing events: skipped cassette exons (SEs), alternative 5′ splice sites (A5SSs), alternative 3′ splice sites (A3SSs), mutually exclusive exons (MXEs) and retained introns (RIs). Segmentation of the significant AS changes by condition (HD versus NT control) revealed that approximately 70% of SE events result in higher exon exclusion in HD in both affected brain regions, cortex and striatum (Fig. 1b and Supplementary Data 4). GO analysis found enrichment for increased exon exclusion in cortical and striatal genes responsible for synaptic transmissions in neurons (Supplementary Fig. 1h,i). Consistent with AS events contributing to gene expression alteration, approximately 60% of the genes with AS are upregulated or downregulated in the cortex and striatum of R6/2 mice (Supplementary Fig. 1j). We then employed a splicing-specific sequencing strategy (RNA-mediated oligonucleotide annealing, selection and ligation with next-generation sequencing (RASL-seq))^61,62^ with 9,496 primer pairs targeting known splice junctions in the cortex of HD R6/2 and two additional knock-in HD mouse models expressing 150 repeats (Q150)^54,55^ or 175 repeats (Q175)^56^ at 3 months, 7 months and 18 months, respectively. Consistent with our RNA-seq and rMATS analysis findings, RASL-seq identified an increase in exon exclusion events in 3mos R6/2 compared to NT littermates (Fig. 1c). Furthermore, we observed an mHTT dose-dependent and age-dependent increase in exon exclusion in the Q150 and Q175 mice (Fig. 1c). Validation of splicing alterations identified by RASL-seq was performed using semi-quantitative RT–PCR for genes that overlapped (*Mtap7d2*, *Mag* and *Mlx*) among the three mouse models (Supplementary Fig. 2a,b; uncropped blots in Supplementary Fig. 10). Finally, we explored if the higher exon exclusion events translated to human subjects. We carried out rMATS analysis of two published HD RNA-seq datasets from the prefrontal cortex (Brodmann area (BA) 9; grade III/IV, 20 HD, 49 controls) and anterior cingulate cortex (grade III/IV, six HD, six controls)^63^. In total, 12,469 AS events were detected in the BA9 samples and 7,730 in the anterior cingulate cortex samples (FDR < 0.05). We observed higher exon exclusion events in patients with HD, confirming that increased exon exclusion is a prominent AS signature in human HD (Fig. 1b).

### Splicing validated by long-read and single-nuclei sequencing

We validated the increase in HD exon exclusion events using single-nuclei RNA-seq and long-read sequencing (Iso-seq)^64^. The RNA-seq data do not account for the cellular heterogeneity of the striatum; therefore, we determined whether there is a cell type specificity for the observed splicing signature. We previously carried out single-nuclei RNA-seq on the striatum and cortex from a different cohort of male HD R6/2 and NT animals at the same symptomatic 3mos stage^65^. Splicing analysis of this dataset using DESJ-detection^66^ showed that most of the splicing alterations were arising in the inhibitory MSNs, the cell type most affected in HD (Fig. 1d). We also employed long-read sequencing using the Pacific Biosciences (PacBio) Iso-seq platform as a high-throughput method to validate our detected splicing changes by sequencing full-length mRNA-enriched transcripts as described^64^. From a subset of the samples described above for short-read RNA-seq (*n* = 4 per condition, males only), we captured 56,000 isoforms from the cortex and 53,000 isoforms from the striatum. We focused our analysis on this splicing event because we detected the most significant AS differences for exon exclusion. Long-read sequencing identified 1,840 of 2,311 (79.6%) genes with significant exon exclusions detected by short-read sequencing in the cortex of R6/2 mice and 2,083 of 2,634 (79.1%) significant exon exclusions in the striatum (Fig. 1e).

### TDP-43 and m6A motifs are enriched in HD R6/2 excluded exons

Because an enriched number of HTT interactors are RBPs, we investigated whether the presence of mHTT may alter RNA splicing by disrupting RBP interactions with their RNA targets. We used HOMER^67^ to perform a de novo motif analysis for the excluded exons affected in HD. The analysis revealed modest but significant enrichment for the UGUGU and UGACU RNA sequence motifs (Fig. 1b). UG/GU-rich motifs are RBP preference sites for TAR DNA-binding protein (*TARDBP*/TDP-43)^23,68,69^. HOMER analysis also revealed that a second potential regulatory binding motif is the canonical m6A DRACH motif (D = A/G/U, R = A/G, H = A/C/U; including UGACU) recognized by writer proteins such as METTL3 (refs. ^70,71^) (Fig. 1f). Loss of TDP-43 is associated with downregulation of long intron-containing genes^22,23^. Consistent with a disruption of TDP-43 in HD mice, transcripts downregulated in the cortex and striatum of R6/2 mice have, on average, longer introns than upregulated genes (Supplementary Fig. 3a). TDP-43 autoregulates its own transcript by binding its 3′ untranslated region (UTR), and loss of nuclear TDP-43 protein results in a modest increase of *TDP-43* mRNAs^23,72^. Analysis of *TDP-43* RNA expression level by RNA-seq in R6/2 mice (Supplementary Fig. 3b) revealed a modest increase in *TDP-43* RNA levels, consistent with a loss of nuclear TDP-43.

We next compared the HD R6/2 excluded exons to splicing alterations identified in mouse NSC34 neuronal cells with small interferring RNA (siRNA)-mediated depletion of TDP-43 (ref. ^73^) and from wild-type mice treated with intracerebroventricular (ICV) injection of TDP-43 antisense oligo (ASO) or control (*n* = 4–5 per treatment). We observed a 43.5% and 66% gene overlap in excluded exons, respectively, with approximately 50% in the same direction (Supplementary Fig. 3c). Our data suggest that TDP-43 contributes to transcriptional dysregulation in the HD R6/2, which may involve an exciting intersection between TDP-43 and m6A in HD that has not been previously described. Therefore, we investigated how TDP-43 and the m6A RNA modification might contribute to altered splicing and HD pathology.

### Unannotated splicing events are observed in HD systems

**Fig. 2:**
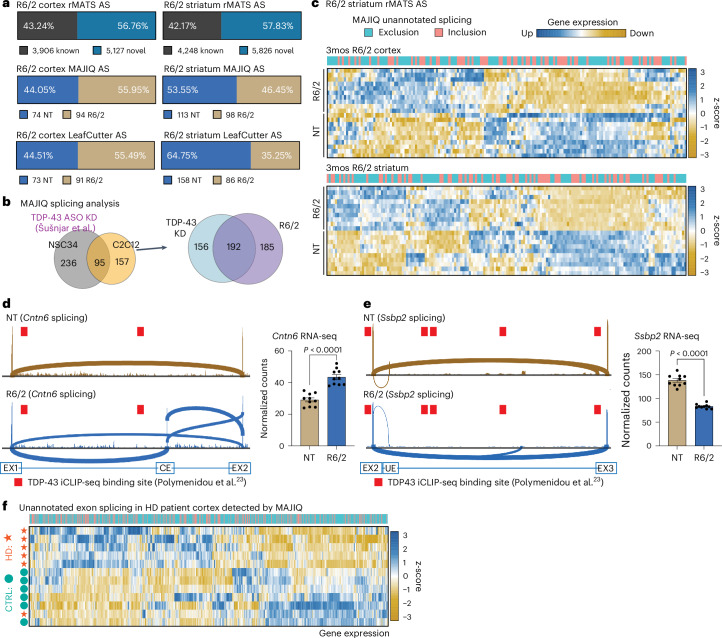
Unannotated splicing events in HD drive gene expression changes. **a**, Bar plot showing rMATS splicing changes that are annotated (known) versus not annotated (novel). Bar plots showing MAJIQ and LeafCutter output for the detection of novel unannotated splicing events in the cortex and striatum from 3mos R6/2 and NT. **b**, Venn diagram showing MAJIQ junction overlap between TDP-43 KD changes from Šušnjar et al.^73^ versus R6/2 cortex and striatum samples. **c**, Heatmap showing exclusion and inclusion of novel unannotated splicing events in 3mos HD R6/2 versus NT (cortex and striatum). Gradient scale represents z-scores of normalized gene counts. **d**,**e**, Integrated Genomics Viewer^99^ sashimi plots of increased CE splicing in R6/2 (*Cntn6*; **d**) or loss of novel unannotated exon (UE) in NT (*Ssbp2*; **e**) in R6/2 and corresponding gene expression changes of the genes that contain the splicing event with statistical significance determined by unpaired two-tailed *t*-test (*Cntn6*: *P* < 0.0001, *t* = 7.111, degrees of freedom (d.f.) = 18, *F* = 1.612, 95% confidence interval (CI): 10.13–18.64; *Ssbp2*: *P* < 0.0001, *t* = 13.35, d.f. = 18, *F* = 6.684, 95% CI: −63.21 to −46.02). Data are presented as mean values ± s.e.m. *n* = 10 biological replicates per genotype (five males, five females). iCLIP-seq, individual-nucleotide resolution UV cross-linking and immunoprecipitation followed by high-throughput sequencing. **f**, Heatmap showing exclusion and inclusion of novel unannotated splicing events detected by MAJIQ in HD patient cortex compared to non-HD control from Al-Dalahmah et al.^63^ and corresponding gene expression changes of the genes that contain the splicing event. Gradient scale represents z-scores of normalized gene counts. CTRL, control.

TDP-43 acts as a repressor of CEs in ALS/FTD, with TDP-43 nuclear loss resulting in increased inclusion of CEs^33–36,74,75^. We initially performed rMATS analysis with the novel splicing detection option^60^. Separating annotated AS events from unknown unannotated events, approximately 50% of the AS changes were novel in the R6/2 cortex and striatum (Fig. 2a), not having been previously reported for these brain regions. We next applied the approach from Brown et al.^34^ and Ma et al.^35^ for novel splicing detection by subjecting our data to MAJIQ and LeafCutter analysis^76,77^. MAJIQ detected 168 (74 NT, 94 R/62) novel cortical AS events and 211 (113 NT, 98 R/62) striatal events, after applying the probability cutoff of >0.90 and *P*(deltaPSI > 0.1) for discovery (Fig. 2a). LeafCutter detected 164 (73 NT, 91 HD) cortical and 244 (158 NT, 86 HD) striatal novel events after using an adjusted *P* value cutoff of <0.05 and deltaPSI > 0.1 (Fig. 2a and Supplementary Data 5). GO analysis revealed that the genes with novel splicing changes occur in the following categories: modulation of excitatory postsynaptic potential, regulation of cation channel activity, neuromuscular junction development, regulation of dendritic spine morphogenesis and positive regulation of neuron projection development (Supplementary Fig. 3d). Using long-read sequencing from the same samples, we found evidence of approximately 57% of the novel splice junctions (Supplementary Fig. 3e). Comparing the novel splicing events from R6/2 to splicing events identified in mouse motor neuron-like (NSC34) cells and myoblast (C2C12) cells with TDP-43 knockdown (KD) from Šušnjar et al.^73^, we observed 55% overlap of TDP-43-dependent splicing changes (192/348) (Fig. 2b). Inclusion of CEs often results in reduced levels of transcripts due to nonsense-mediated decay or premature polyadenylation^33–37^; however, the novel splicing events observed in the R6/2 model corresponded to expression changes in both directions (Fig. 2c). Splicing sashimi plot examples of differential unannotated exon usage are shown in Fig. 2d,e. For example, for *Cntn6*, the exon arises in R6/2, with a corresponding increased RNA expression, whereas, for *Ssbp2*, the unannotated exon is not present in R6/2, with a corresponding decreased RNA expression. Analysis of CLIP-seq data^23^ shows that TDP-43 binding sites are present within the intron containing the unannotated exons, supporting a direct role for TDP-43 in regulating these splicing events. Our analysis identified altered TDP-43 localization and splicing changes in the R6/2 mouse model in genes responsible for neuronal development and function that are dysregulated in HD. We next set out to understand whether previously unannotated splicing events are altered in human HD. Through MAJIQ analysis, we detected 78 events (42 controls, 36 HD) in the prefrontal cortex^78^ and 118 events (122 controls, 136 HD) in the anterior cingulate cortex^63^. Similar to R6/2 mice, the unannotated splicing changes in the human data contribute to DGE changes in both directions (Fig. 2f). Therefore, the human HD RNA-seq analysis confirms the findings in mice of increased exon exclusion events and altered, previously unannotated, splicing in HD that can result in both increased and decreased expression and/or RNA stability.

### TDP-43 binding is attenuated in dysregulated HD R6/2 genes

**Fig. 3:**
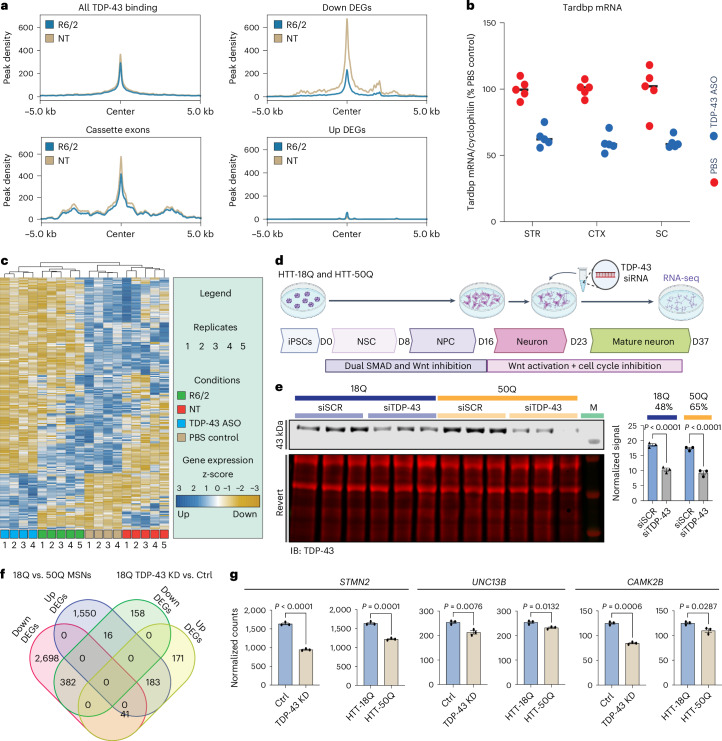
TDP-43 knockdown corresponds to R6/2 transcriptional changes. **a**, Reproducible R6/2 HD and NT IDR TDP-43 peaks were centered and plotted on all TDP-43 binding sites, compared to Down DEGs, Up DEGs and cassette exons. **b**, *TDP-43* mRNA level by qPCR normalized to cyclophilin as a percentage of PBS control. CTX, cortex; SC, spinal cord; STR, striatum. *n* = 5. Black bar represents the mean. **c**, Heatmap showing clustering of 3mos HD R6/2, NT, TDP-43 ASO treated and control PBS treated on TDP-43 KD-dependent DGE changes. Gradient scale represents z-scores of normalized gene counts. **d**, Schematic of iPSC differentiation into MSNs with TDP-43 KD by siRNA. D, day; NPC, neural progenitor cell; NSC, neural stem cell. **e**, Left: western blot for TDP-43 protein levels after treatment of MSNs with TDP-43 siRNA. *n* = 3 differentiation replicates per condition. Right: bar graph plots TDP-43 intensity normalized to Revert total protein stain. Data are presented as mean values ± s.e.m. Statistical significance was determined by two-way ANOVA with Sidak’s multiple comparisons test (18Q: *P* < 0.0001, 95% CI: 6.230–10.20; 50Q: *P* < 0.0001, 95% CI: 6.084–10.06). IB, immunoblot; M, marker. **f**, Venn diagram showing the overlap of DEGs between HTT-18Q MSNs scramble control versus TDP-43 siRNA and HTT-18Q MSNs versus mHTT-50Q. **g**, Example of key gene expression changes anticipated from TDP-43 KD. *n* = 3 differentiation replicates per condition. Statistical significance was determined by unpaired two-tailed *t*-test. Data are presented as mean values ± s.e.m. (TDP-43 KD versus Ctrl *STMN2*: *P* < 0.0001, *t* = 24.41, d.f. = 4, *F* = 2.639, 95% CI: −768.6 to −611.6; 18Q versus 50Q *STMN2*: *P* = 0.0001, *t* = 14.49, d.f. = 4, *F* = 2.344, 95% CI: −496.4 to −336.7; TDP-43 KD versus Ctrl *UNC13B*: *P* = 0.0076, *t* = 4.988, d.f. = 4, *F* = 2.598, 95% CI: −64.21 to −18.29; 18Q versus 50Q *UNC13B*: *P* = 0.0132, *t* = 4.242, d.f. = 4, *F* = 2.249, 95% CI: −36.78 to −7.682; TDP-43 KD versus Ctrl *CAMK2B*: *P* = 0.0006, *t* = 10.04, d.f. = 4, *F* = 5.482, 95% CI: −47.26 to −26.79; 18Q versus 50Q *CAMK2B*: *P* = 0.0287, *t* = 3.345, d.f. = 4, *F* = 4.953, 95% CI: −29.00 to −2.693). Ctrl, control.

To further investigate the impact of TDP-43 on RNA metabolism in HD, we focused on RNA targets of TDP-43. To identify TDP-43 RNA targets that may be altered by the presence of mHTTex1, we carried out TDP-43 eCLIP-seq^79^ on HD R6/2 and NT mouse cortex and striatum (*n* = 8; age = 3 months; four males, four females) (Supplementary Fig. 4a). Surprisingly, our binding site distribution primarily spanned coding regions and 3′ UTR, unlike previous findings^23,68^ that showed primarily intronic binding (Supplementary Fig. 4b). This difference could be due to technical aspects of our experiment that enriched for cytoplasmic processed mRNAs rather than intron-containing nuclear RNAs. Analysis of 5mer U-rich motifs in our TDP-43 CLIP peaks from these coding regions revealed an enrichment for UGUGU, as previously reported (Supplementary Fig. 4c). The TDP-43 eCLIP-seq yielded approximately 60% target gene overlap with our reanalysis of Polymenidou et al.^23^. There is an approximately 80% overlap between HD R6/2 and NT TDP-43 binding to RNA transcripts in both brain regions, suggesting that most targets remain unchanged due to mHTTex1 expression (Supplementary Fig. 4d). Mapping TDP-43 significant peaks to genes with excluded exons identified from HD R6/2 cortex and striatum (Fig. 1b) revealed TDP-43 binding on approximately 57–64% of genes containing exon exclusions (Supplementary Fig. 4e). Next, we assessed the possibility that TDP-43 drives HD R6/2 transcriptional dysregulation through direct binding to genes constituting the striatal HD signature. TDP-43 binds approximately 30% (79/266) of HD266ssg, most downregulated in HD (Supplementary Fig. 4f). TDP-43 binding density was found to be decreased in R6/2 mice on downregulated genes and transcripts with abnormal splicing of cassette exons but not on transcripts that are upregulated (Fig. 3a). This suggests that loss of TDP-43 binding to target genes can decrease their expression and promote cassette exon splicing.

We reasoned that if TDP-43 is a regulator of HD gene expression, ASO-mediated depletion of TDP-43 should recapitulate, at least partially, gene expression changes similar to HD. Therefore, we carried out RNA-seq from the striatum of wild-type mice treated with an intraventricular injection of ASOs against TDP-43 or PBS (*n* = 4 per treatment). The approach yielded approximately 50% downregulation of TDP-43 2 weeks after injection as demonstrated by qRT–PCR in the cortex, striatum and spinal cord (Fig. 3b). Heatmap hierarchical clustering of TDP-43 KD-dependent DEGs revealed clustering of 3mos R6/2 HD animals with TDP-43 ASO-treated animals (Fig. 3c). We next tested siRNA-mediated TDP-43 KD in isogenic induced pluripotent stem cell (iPSC) lines expressing non-pathogenic length HTT-18Q or HD causing mHTT-50Q and differentiated into MSNs^80^ (Fig. 3d). Differentiation efficiency was determined by immunofluorescence (IF) for DARPP32, CTIP2, FOXP1, FOXP2 and MAP2 (Supplementary Fig. 4g). After confirmation of TDP-43 protein KD by western blot analysis (Fig. 3e; uncropped blots in Supplementary Fig. 11), samples were subjected to RNA-seq. Our analysis revealed 4,870 DEGs (3,121 downregulated, 1,749 upregulated) when comparing HTT-18Q versus mHTT-50Q (Supplementary Data 6; ‘genotype’ effect). For the ‘TDP-43 KD’ effect, we observed 951 DEGs (556 downregulated, 395 upregulated), comparing HTT-18Q TDP-43 KD versus scramble siRNA control (Supplementary Data 7). We applied the hypergeometric test and observed that 68.7% (382/556, *P* = 1.5 × 10^−189^) of the TDP-43 KD downregulated transcripts overlapped with HD downregulated transcripts, and 46.3% (183/395, *P* = 7.5 × 10^−93^) overlapped with the HD upregulated genes (Fig. 3f and Supplementary Data 8). Although we did not observe abnormal CEs for *STMN2*, we did observe downregulation of *STMN2*, *UNC13B* and *CAMK2B* expression in both HD and TDP-43 KD iPSC-derived MSNs (Fig. 3g). Together, our analysis shows that TDP-43 KD drives similar gene expression pattern changes as mHTT in HD MSNs.

### Reduced nuclear TDP-43 in HD mouse and human brains

**Fig. 4:**
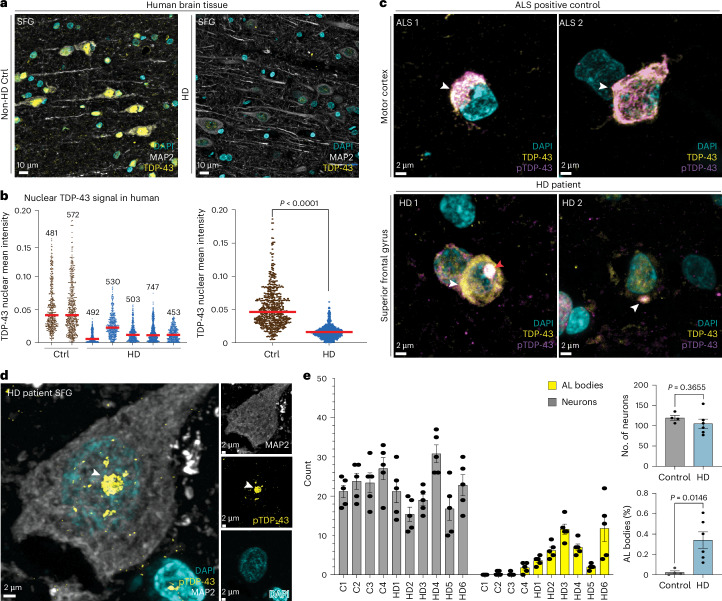
TDP-43 protein mislocalizes in HD patient brain. **a**, Representative IF staining images of SFG from patients with HD compared to non-HD control individuals showing decreased TDP-43 (yellow) signal intensity. **b**, Left: quantification of decreased nuclear TDP-43 signal intensity; five representative images were taken at ×40 from five HD and two control individuals. A CellProfiler pipeline was created to identify larger nuclei (enriched for neurons) by DAPI staining. The average of TPD-43 nuclear signal was obtained by measuring the intensity signal within a mask defined by DAPI. Each cell’s mean nuclear TDP-43 intensity is plotted. One-way ANOVA was performed with multiple comparisons and resulted in significant changes between all HD versus control comparisons (data not shown). Numbers on top of each group indicate the number of cells plotted. Right: dot plot showing grouped data by genotype. Statistical significance was derived from unpaired two-tailed *t*-test between control versus HD (*P* < 0.0001, *t* = 31.41, d.f. = 1317, *F* = 10.33, 95% CI: −0.03850 to −0.03397). Red bar represents the median. **c**, Representative IF staining images of the motor cortex from a patient with ALS (positive control) compared to patients with HD, using antibodies against total TDP-43 (yellow), phosphorylated TDP-43 (purple) and nuclear stain DAPI. White arrowhead indicates pTDP-43 cytoplasmic aggregation; red arrowhead (HD1) indicates cytoplasmic aggregate verified by orthogonal view. **d**, IF images showing pTDP-43 AL bodies (yellow) within MAP2-positive neurons (white). **e**, Bar graph showing counts of DAPI, MAP2-positive and AL bodies. Data are presented as mean values ± s.e.m. Each bar is derived from five random ×20 images from each patient. C, control; HD, patient with Huntington’s disease. %AL bodies is the number of AL bodies per patient normalized to the total number of Map2-positive neurons. Statistical significance was determined by unpaired two-tailed *t*-test (number of neurons: *P* = 0.3655, *t* = 0.9593, d.f. = 8, *F* = 5.329, 95% CI: −48.51 to 20.01; AL bodies (%): *P* = 0.0146, *t* = 3.103, d.f. = 8, *F* = 44.539, 95% CI: 0.08172–0.5545). Experiments in **a**–**e** were repeated at least three times with similar results represented above.

We assessed the protein expression of TDP-43 by IF in cortex from 3mos late symptomatic R6/2 mice and observed decreased nuclear TDP-43 protein expression (Supplementary Fig. 5a). Similar TDP-43 nuclear reduction was observed in cortex from 24mos homozygous Q175 knock-in mice (Supplementary Fig. 5a). Quantification using CellProfiler showed a significant decrease in nuclear TDP-43 levels in both 12mos and 24mos Q175 animals (Supplementary Fig. 5b). This reduced nuclear staining of TDP-43 did not result from reduced TDP-43 protein levels, as quantitative western blot analysis showed equivalent TDP-43 levels in HD R6/2 cortex (Supplementary Fig. 5c; uncropped blots in Supplementary Fig. 9a). TDP-43 is one of the RBPs listed as an interactor of HTT protein^13,19,20^, including by proteomic analysis^71^. We confirmed this interaction by co-immunoprecipitation (co-IP) of TDP-43 from mouse brain with mouse full-length HTT (FL-HTT) (Supplementary Fig. 5d; uncropped blots in Supplementary Fig. 9b). Decrease of nuclear TDP-43 was also found in human HD postmortem superior frontal gyrus (SFG) compared to tissue from unaffected individuals by IF staining (control: *n* = 2; HD: *n* = 5) (Fig. 4a and Supplementary Fig. 5e). Quantification of nuclear TDP-43 levels using CellProfiler revealed a significant decrease in nuclear TDP-43 fluorescent intensity in all five patients with HD compared to non-HD tissues (Fig. 4b), supporting loss of TDP-43 function as a contributor to HD pathogenesis. To examine nuclear-cytoplasmic translocation and phosphorylation of TDP-43 (pTDP-43), we co-stained brain tissues from HD and unaffected individuals with a TDP-43 antibody and a pTDP-43 antibody specific to phosphorylated serine 409 and serine 410 (S409/S410) by IF. We detected the presence of rare cytoplasmic TDP-43 aggregates, recognized by both antibodies in patients with HD but not in unaffected individuals (Fig. 4c). Furthermore, the aggregate-like morphology was localized to the perinuclear space, similar to but less dense and fibrous than pTDP-43 inclusions in ALS/FTD^21,22,24,26^ or in ALS motor cortex evaluated (separated channels in Supplementary Fig. 6a; low magnification in Supplementary Fig. 6b).

### Phosphorylated TDP-43 and HTT in intranuclear bodies

In addition to cytoplasmic accumulation of pTDP-43, and co-localization of TDP-43 with nuclear HTT inclusions, we observed a spherical accumulation of pTDP-43 in the nucleus of Map2-positive neurons. These structures are an average of approximately 3 µm in diameter and are extremely rarely detected in the control human tissues (Fig. 4d and Supplementary Fig. 7a). We refer to these structures as pTDP-43 nuclear aggregation-like bodies (AL bodies). In HD patient tissue, pTDP-43 AL bodies contained N-terminal TDP-43 (Supplementary Fig. 7b) and HTT (Ab 5526) (Supplementary Fig. 7c). A second S409/S410 pTDP-43 antibody produced similar localization to AL bodies (Supplementary Fig. 7d). Notably, pTDP-43 and HTT protein co-localize in AL bodies, but do not co-localize in cells with canonical HTT nuclear inclusions (Supplementary Fig. 7e). Quantification of the percentage of Map2-positive neurons with AL bodies in four control and six patients with HD revealed a significant increase of AL bodies in HD brains compared to controls (Fig. 4e). This striking accumulation of nuclear pTDP-43 and HTT into distinct spherical AL bodies in MAP2-positive neurons from HD patient brains represents a type of TDP-43 pathology not previously described that may be unique to HD.

### m6A sequencing reveals an altered epitranscriptome in HD R6/2 mice

**Fig. 5:**
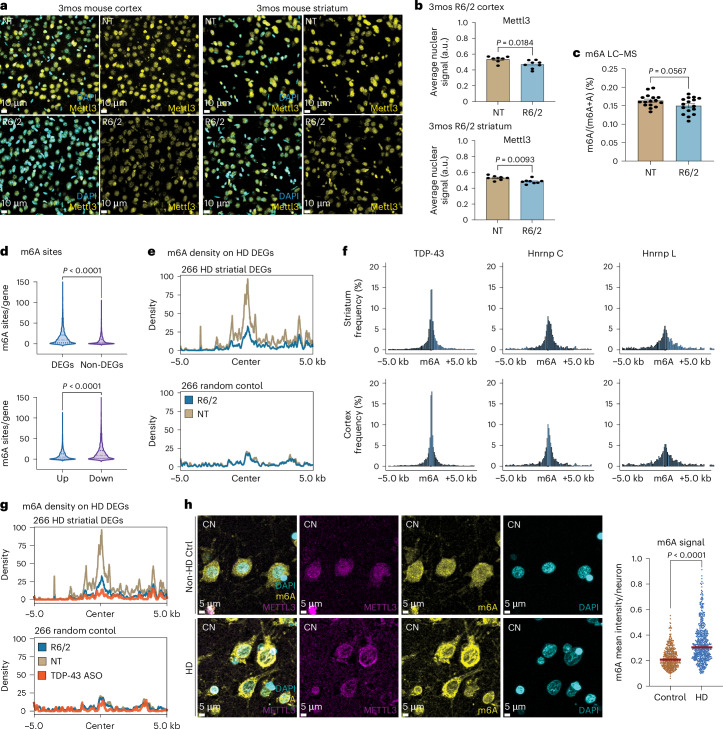
m6A is dysregulated in HD. **a**, Representative images showing modest decrease in Mettl3 signal in the R6/2 cortex and striatum. **b**, CellProfiler analysis of Mettl3 nuclear intensity; statistical significance was determined by unpaired two-tailed *t*-test (cortex: *P* = 0.0184, *t* = 2.693, d.f. = 13, *F* = 1.572, 95% CI: −0.1158 to −0.01270; striatum: *P* = 0.0093, *t* = 3.052, d.f. = 13, *F* = 1.044, 95% CI: −0.0774 to −0.0324). *n* = 8 (four males, four females) per genotype were quantified, each averaging three cortical or striatal regions. Data are presented as mean values ± s.e.m. **c**, MS for m6A showed no significant changes by unpaired two-tailed *t*-test (*P* = 0.0567, *t* = 1.982, d.f. = 30) in global m6A levels. Data are presented as mean values ± s.e.m. *n* = 16 per genotype (five males, 11 females). **d**, Violin plots showing increased m6A sites per gene on genes that are dysregulated in HD R6/2 (DEGs), in particular in downregulated genes compared to upregulated genes. Statistical significance was determined by unpaired two-tailed *t*-test. DEGs versus non-DEGs: *P* < 0.0001, *t* = 15.03, d.f. = 3818, *F* = 3.099, 95% CI: −9.075 to −6.981; Up versus Down: *P* < 0.0001, *t* = 11.52, d.f. = 3384, *F* = 2.870, 95% CI: 5.688–8.021. **e**, m6A site deposition (localized levels) on 266 genes dysregulated in R6/2 striatum. **f**, Histogram plot showing TDP-43 eCLIP sites relative to an m6A site (from m6A eCLIP data) (0 position) compared to Hnrnp C and Hnrnp L binding motifs. **g**, m6A site deposition (localized levels) on 266 genes dysregulated with TDP-43 KD by ASO. **h**, Representative images showing significant increase in m6A IF intensity in HD patient caudate nucleus (CN) with corresponding METTL3 staining. Dot plot with each dot representing the average m6A intensity per neuronal cell spanning five ×20 confocal images per patient (three controls, six HD). Samples were grouped to either control or HD group with the unpaired Studentʼs *t*-test for significance (for separated dot plots, see Supplementary Fig. 7g) (*P* < 0.0001, *t* = 13.82, d.f. = 956, *F* = 3.313, 95% CI: 0.09684–0.1289). Bar represents the median. Experiments in **a**, **b** and **h** were repeated at least three times with similar results represented above.

Recognizing that RNA-seq analysis (Fig. 1g) identified a potential regulatory program in HD from the presence of the canonical m6A motif in dysregulated RNAs, we set out to examine (1) pathological evidence of m6A dysfunction and (2) how the HD epitranscriptome may contribute to AS changes and pathogenesis. First, we assessed the proteins of the m6A machinery, both m6A writers (Mettl3, Mettl14, Rbm15 and Wtap) and erasers (Alkbh5 and Fto), in the cortex and striatum from HD R6/2 at 3mos (late symptomatic) (*n* = 8, males and females). Because the proteins of the m6A machinery are localized to the nucleus, we used CellProfiler to segment out the nuclear signal and quantified the average intensity per cell. Applying the same methodology across all the stained sections, a significant decrease in nuclear expression was identified for Mettl3 in both the cortex and striatum (Fig. 5a,b) and for Rbm15 in the cortex, whereas the other m6A machinery proteins were unchanged (Supplementary Fig. 7a). With a reduction in Mettl3 protein expression, m6A levels are expected to be decreased in HD R6/2 mice. However, liquid chromatography–mass spectrometry (LC–MS) analysis of polyA-selected RNA from mouse cortex (*n* = 16 per genotype (five males, 11 females)) samples did not show a significant global change of m6A levels (Fig. 5c). Because the global LC–MS analysis cannot capture gene-level m6A changes, we carried out single-nucleotide-resolution, transcriptome-wide m6A sequencing using eCLIP-seq with an m6A-specific antibody^79^. m6A eCLIP-seq identified 28,8976 shared, 5,273 HD and 3,035 NT m6A genomic locations in the cortex and 30,459 shared, 3,355 HD and 3,905 NT in the striata, respectively (Supplementary Fig. 8b). Motif enrichment revealed that more than 70% of the detected m6A peaks fall within the expected DRACH m6A motif as previously reported (Supplementary Fig. 8c) and recapitulate the concentrated distribution of m6A sites at the 3′ UTR in a metagene plot (Supplementary Fig. 8d). We then determined whether m6A deposition can influence the HD R6/2 transcriptome by evaluating enrichment for m6A deposition on HD R6/2 DEGs. After normalizing for length, genes that comprise DEGs have significantly more m6A sites than non-DEGs (Fig. 5d), and those genes that are downregulated in the HD R6/2 have a greater number of m6A sites (Fig. 5d), suggesting that m6A is enriched and may play a regulatory role in the HD R6/2 dysregulated genes. Plotting the m6A sites found on the HD R6/2 DEGs across a metagene plot revealed that the increased m6A sites were enriched for 3′ UTR m6A sites on downregulated DEGs and for coding sequence (CDS) m6A sites on upregulated DEGs (Supplementary Fig. 8e).

TDP-43 binding was decreased in genes defining the striatal HD signature. Similarly, m6A deposition decreased in the same striatal genes in the HD condition (Fig. 5e); for example, whereas genes that are DEGs had the highest number of detected m6A sites, in the HD context the number of transcripts methylated at those sites was decreased. Given the enrichment of the m6A and TDP-43 binding motifs on HD dysregulated genes, we evaluated whether TDP-43 clustered close to m6A sites in the R6/2 mice. Centering the m6A sites and looking 5 kilobases (kb) upstream and downstream, the TDP-43 binding sites were plotted relative to the m6A site at the center position. We next looked for known RBP motifs (Hnrnp C – UUUUU / HnrnpP L – ACACA) as a comparison and controlled for expressed genes in the R6/2 striatum and cortex (Fig. 5f). TDP-43 has a higher center peak overlapping with m6A sites compared to Hnrnp C and Hnrnp L. Hnrnp C is a known m6A reader protein that recognizes the m6A motif. The higher peak of TDP-43 suggests that the binding of TDP-43 may be more dependent on m6A than Hnrnp C in HD^81^ and that a decrease in m6A deposition on dysregulated HD genes results in decreased TDP-43 binding. McMillan et al.^51^ reported that the binding of TDP-43 to RNA depends on m6A. This finding and our results suggest a direct connection between RNA modification status and TDP-43 binding. In the case of HD, m6A modification and TDP-43 binding are associated with mHTT-dependent transcriptomic alterations; genes that tend to be differentially expressed have more m6A sites; however, in HD, those same sites are less methylated. Based on our data and the data from McMillan et al.^51^, we hypothesize that m6A alterations precede TDP-43 dysregulation. Thus, to evaluate whether m6A or TDP-43 are primary regulators of HD gene expression, we performed m6A eCLIP-seq on TDP-43 ASO-treated mice (*n* = 3). A decrease in m6A deposition on HD genes was observed in both R6/2 and TDP-43 ASO-treated mice (Fig. 5g). We ruled out the possibility that the signal is driven by transcript-level differences between R6/2 and NT, as we did not observe higher m6A deposition on upregulated genes in either R6/2 or TDP-43 ASO-treated mice (Supplementary Fig. 8f). Finally, we performed IF staining for METTL3 and m6A in HD patient caudate nucleus (three controls, six HD). We observed a significant increase in the overall m6A signal in HD caudate nucleus (Fig. 5h; individual patient quantification in Supplementary Fig. 8g and lower magnification in Supplementary Fig. 8h), with increased cytoplasmic accumulation of the m6A signal, similar to observations in AD patient brains reported in Jiang et al.^82^. Furthermore, METTL3 is also mislocalized to the cytoplasm (Fig. 5h; lower magnification in Supplementary Fig. 8h). These human data are consistent with the dysregulation of m6A modification in the R6/2 mice. These results support that (1) there is a connection between m6A RNA modification and TDP-43 and (2) TDP-43 dysfunction occurs before m6A dysregulation.

## Discussion

Dysregulation of TDP-43 in ALS and FTLD is an active area of investigation, including the development of biomarkers and therapeutic strategies, such as ASOs, to restore appropriate splicing of TDP-43 targets^83–85^. Recently, TDP-43 function was shown to be influenced by RNA modifications, such as m6A and m1A, with m1A found on CAG-expanded genes^51,86^. Although splicing changes have been documented in HD^87–89^, including the abnormal splicing of *HTT* RNA leading to the expression of Httex1a protein^11,90^, the underlying mechanisms and potential involvement of TDP-43 as a driver of mis-splicing in HD have not been defined. Here we show that TDP-43 loss of function and aberrant m6A modifications contribute to mis-splicing in HD, leading to the altered expression of critical striatal genes known to be dysregulated in HD. Building on our previous report showing altered TDP-43 localization in human patient brain^18^, we generated and integrated multi-omics data, including RNA-seq, RASL-seq, PacBio long-read sequencing, m6A eCLIP-seq and TDP-43 eCLIP-seq, to investigate mechanisms involved in aberrant splicing. We demonstrated that the RBP TDP-43 and the m6A writer METTL3 have altered protein subcellular localization and protein expression, respectively, in R6/2 mice and in human HD brain. These alterations accompanied a corresponding enrichment in HD-specific AS and decreased interaction with dysregulated RNAs defining the striatal HD signature. IF imaging in HD mice and HD patient brain tissue revealed co-localization of TDP-43 with mutant HTT in nuclear inclusions, decreased nuclear TDP-43 and a corresponding increase in aggregated phosphorylated TDP-43 in the cytoplasm. We also found an accumulation of spherical, fibrous-like pTDP-43 in the nucleus of Map2-positive neurons that co-localize with HTT. Our analysis of RNA-seq data from both mouse and human samples revealed changes in AS with increased exon exclusion events in HD.

We observed that 60% of the altered splicing events occurred within genes that are differentially expressed in HD, with most leading to decreased gene expression. We hypothesize that mHTT can disrupt the normal interactions of HTT with RBPs, including TDP-43 and METTL3, which may result in RBPs not being properly bound to their RNA targets, thus leading to dysregulated AS observed in our study. Consistent with this hypothesis, we showed that TDP-43 binding to genes crucial to striatal neuronal maturation and survival is decreased in HD. m6A sequencing revealed increased m6A sites on genes differentially downregulated in HD compared to non-DEGs. However, m6A depositions at specific sites on these genes are decreased in HD. This finding synergizes with the TDP-43 CLIP-seq data, as we report that TDP-43 binding is enriched near m6A modification. Functional crosstalk between RBPs and m6A has been shown for Tau, where HNRNPA2B1 connects oligomeric Tau with m6A modification as a linker^82^. Furthermore, m6A modification is required for TDP-43 binding to RNA in ALS spinal cord^51^, and toxicity is modulated through genetic perturbation of m6A machinery. Our finding that TDP-43 binding corresponds with m6A deposition on downregulated striatal genes in HD suggests a co-regulatory role for m6A modification with TDP-43 in HD.

We also report the expression of previously unannotated exon splicing in both HD mouse and human brain RNA, consistent with disruption of the known function of TDP-43 as a negative regulator of CEs^33,36,91^. Nuclear clearance of TDP-43 and aggregation of phosphorylated TDP-43 in the cytoplasm causes an increase in CE insertion in several transcripts, including *STMN2* and *UNC13a*^33–37,83^. Using isogenic iPSC-derived MSNs, we detected altered novel exon usage in HD neurons similar to the effect of TDP-43 KD in control iPSC neurons. Furthermore, a significant overlap was observed with alterations previously reported in mice with TDP-43 KD^23^. We also observed that the presence of aberrant, previously unannotated exon splicing corresponds to hallmark HD genes that are primarily downregulated; however, unexpectedly, both an increase and a decrease in novel unannotated exon expression were identified to result in DGE. We propose a mechanism in which the interaction of HTT with multiple RBPs regulates CE splicing. Further studies are required to identify additional CE-regulating RBPs.

Detecting nuclear pTDP-43 AL bodies in our study may represent a TDP-43 pathology unique to HD. These circular AL bodies do not morphologically resemble known nuclear aggregates^92^. The co-localization of AL bodies with HTT only in neurons lacking canonical HTT nuclear inclusions suggests the possibility that aggregation of TDP-43 in HD is dependent on mHTT, until mHTT reaches a seeding density to form the canonical nuclear inclusions. The shape of AL bodies suggests an unidentified core, similar to PML bodies, forming a donut-like structure around ubiquitinated and sumoylated proteins^93^. Alternatively, AL bodies may co-localize with the nucleolus. Finally, AL bodies may represent RNA-free TDP-43-containing anisosomes within the nucleus that we previously showed can occur via proteosome inhibition or specific mutation in TDP-43’s RNA recognition motif^94^. Our TDP-43 eCLIP-seq detected decreased TDP-43 binding to RNAs, making it plausible that these AL bodies may be similar to anisosomes and contain RNA-free TDP-43; this can be addressed in future studies. Here, we identified three TDP-43 aggregation phenotypes in HD, one of which has not previously been observed, which presents the possibility that our molecular readouts are resulting from a combinatorial effect of known TDP-43-dependent regulation and regulation not previously described. Our future efforts will be aimed at elucidating the role of AL bodies in HD progression and neurodegeneration.

In summary, we present here a systematic analysis of mechanisms that may contribute to aberrant splicing in HD, including through post-transcriptional RNA processing. Our data support TDP-43 loss of function coupled with altered m6A modification as a mechanism underlying AS/CE usage and critical gene dysregulation implicated in HD. Neuropathologically, we show an aggregate-like nuclear body in human HD that is independent of classical nuclear inclusions. Finally, the body of work suggests that the impact of TDP-43 dysregulation involved in disease pathogenesis is broad and may represent a pivotal therapeutic target for neurodegenerative diseases in general. The relationship discovered in the present study between TDP-43 and m6A also suggests that emergent methods to control m6A could have a therapeutic benefit through the regulation of TDP-43 (refs. ^95–97^).

## Methods

### Statistics and reproducibility

No statistical methods were used to pre-determine sample sizes; however, sample size selections were made to be similar to previously published studies. All individual data points were shown, and all experiments were repeated at least three times with similar results as represented in this study. Data were assumed to be normally distributed, but this was not formally tested. Where appropriate, all treatment groups were randomly selected, and analyses were performed blinded to treatment/disease status. Data were excluded only for animals that did not survive to endpoint, as per ethical guidance. Differential expression statistics were performed within cited packages below. Statistical testing was performed in GraphPad Prism 10 (GraphPad Software).

### Postmortem brain tissue and animal models

Human brain samples were obtained in collaboration with the Netherlands Brain Bank (NBB), the Netherlands Institute for Neuroscience, Amsterdam (open access: https://www.brainbank.nl/) and the Neurological Foundation of New Zealand Human Brain Bank (NZBB). Information on patients can be found in Supplementary Data 9. All materials have been removed of any patient identifiers. All material was collected from donors for whom or from whom a written informed consent for a brain autopsy and the use of the material and clinical information for research purposes had been obtained by the NBB and the NZBB, approved by the Health and Disability Ethics Committee (ethics no.: 14/NTA/208/AM02), Ministry of Health, New Zealand. Animal experiments were carried out in accordance with the National Institutes of Health Guide for the Care and Use of Laboratory Animals and an approved animal research protocol by the Institutional Animal Care and Use Committee (IACUC, no. AUP-21-087) at the University of California, Irvine (UCI), an institution accredited by the Association for Assessment and Accreditation of Laboratory Animal Care. For RASL-seq, all procedures were conducted in accordance with the guidelines of the University of California, San Diego IACUC (no. S0022). iPSC work carried out in this study was approved by the UCI Human Stem Cell Research Oversight Committee (UCI hSCRO no. 118) and the UCI Institutional Review Board (UCI IRB no. 2008-6556).

### Animal model, handling and tissue harvest

All mice (B6CBA-Tg(HDexon1)62Gpb/3J (no. 006494), B6.129P2-Htttm2Detl/150J (no. 004595), B6J.129S1-Htttm1Mfc/190ChdiJ (no. 027410) and C57BL/6J (no. 000664)) in this study were obtained from The Jackson Laboratory at approximately 5 weeks of age. The sex of animals was balanced and age matched. All mice were housed on a 12-h light/dark schedule with ad libitum access to food and water. Animals were housed at controlled temperature and humidity: 70 ± 2 °F and 50 ± 5% humidity (RASL-seq: (68–79 °F) and humidity (30–70%)). Animals were aged and then euthanized with Euthasol overdose (pentobarbital sodium and phenytoin sodium). Cardiac perfusion was performed with 0.01 M PBS, followed by brain harvesting and isolation of striatum and cortex from the left hemisphere that was flash frozen in liquid nitrogen and stored at −80 °C until use for biochemical analysis. The other halves were post-fixed in 4% paraformaldehyde, cryoprotected in 30% sucrose and cut at 30 μm on a sliding vibratome for immunohistochemistry, as described below. For biochemistry, frozen tissues were lysed (lysis buffer: 50 mM Tris-HCl pH 7.4, 100 mM NaCl, 1% NP-40 (Igepal, CA630), 0.1% SDS, 0.5% sodium deoxycholate, 1:200 Protease Inhibitor Cocktail III (add fresh), 0.1 mM PMSF, 25 mM NEM, 1.5 mM aprotinin, 23.4 mM leupeptin). In brief, samples were homogenized by douncing in lysis buffer followed by incubation on ice for 30 min. Lysate was then sonicated 3× for 10 s at 40% amplitude. Protein quantification was performed by Lowery protein assay with linear range dilution.

### Knockdown of TDP-43 with ASOs in mice

C57Bl/6 males were dosed at 5 weeks of age with PBS control (*n* = 5) or ASO (Ionis Pharmaceuticals) targeting TDP-43 (*n* = 5) at 500 µg by ICV bolus injection. Two weeks after dose, cortex and striatum were collected for downstream analysis.

### Differentiation of iPSCs to MSNs and siRNA knockdown of TDP-43

iPSCs (CS83iCTR33-n1 (RRID: CVCL_IW28)) were derived from fibroblasts from a non-affected patient from the HD iPSC Consortium, 2017 (ref. ^100^) using non-integrating reprogramming techniques and CRISPR modified to an isogenic series with 18Q and 50Q CAG repeat length. Cells were grown on Matrigel-coated plates in tissue culture sterile conditions and passaged with non-enzymatic dissociation medium. Cells were fed with mTeSR1 (STEMCELL Technologies) every day and passaged at approximately 70% confluency. Small-molecule-based differentiation to MSNs was followed by Smith-Geater et al.^80^ and outline in Fig. 3b. In brief, iPSC colonies switched to neural induction medium (Advanced DMEM/F12 (1:1) supplemented with 2 mM GlutaMAX, 2% B27 without vitamin A, 10 μM SB431542, 1 μM LDN 193189, 1.5 μM IWR1) with daily medium changes. On day 4, cells were passaged 1:2 with Accutase and replated on Matrigel. Cells were passaged again 1:2 at day 8 with Accutase and replated on Matrigel in a different medium (Advanced DMEM/F12 (1:1) supplemented with 2 mM GlutaMAX, 2% B27 without vitamin A, 0.2 μM LDN 193189, 1.5 μM IWR1, 20 ng ml^−1^ Activin A). At day 16, cells were dissociated with Accutase and plated at a density of 111,000 per cm^2^ on NUNC-treated tissue culture plastic treated with poly-d-lysine and Matrigel in SCM1 medium (Advanced DMEM/F12 (1:1) supplemented with 2 mM GlutaMAX, 2% B27, 10 μM DAPT, 10 μM forskolin, 300 μM GABA, 3 μM CHIR99021, 2 μM PD 0332991, to 1.8 mM CaCl_2_, 200 μM ascorbic acid, 10 ng ml^−1^ BDNF), 50% medium change every 2–3 d. Full medium change to SCM2 medium at day 23 (Advanced DMEM/F12 (1:1): Neurobasal A (50:50) supplemented with 2 mM GlutaMAX, 2% B27, 1.8 mM CaCl_2_, 3 μM CHIR99021, 2 μM PD 0332991, 200 μM ascorbic acid, 10 ng ml^−1^ BDNF) was carried out, 50% medium change every 2–3 d until day 37. Human TDP-43 and non-targeting control siRNA were obtained from Horizon Discovery (Accell SMARTPool, E-012394-00-0050); cells were treated with siRNA on day 23 and harvested at day 37.

### Co-IP/immunoblotting

For each tissue type, the optimal protein concentration and primary antibody concentration were determined by linear range according to LI-COR’s protocol. For co-IP, 1 mg of protein was used for each IP—1:1,000 dilution of primary antibody and 30 µl of Dynabeads sheep anti-rabbit or goat anti-mouse. IP was carried out by incubation for 1 h at room temperature, and then a wash was performed with 3× high-salt wash buffer (50 mM Tris-HCl pH 7.4, 1 M NaCl, 1 mM EDTA, 1% NP-40, 0.1% SDS, 0.5% sodium deoxycholate), followed by 2× low-salt wash buffer (20 mM Tris-HCl pH 7.4, 10 mM MgCl_2_, 0.2% Tween 20). Elution was achieved by 10-min incubation at 80 °C in 1× LDS and 1 mM DTT. Co-IP and regular western blot samples were run on 4–12% Bis-Tris gels and 3–8% Tris-acetate. Specific protein band analysis was performed using LI-COR Empira Studio software with normalization to Revert total protein stain.

### IF staining

A list of primary and secondary antibodies with dilutions used in this study can be found in Supplementary Data 10.

For mouse samples, coronal sections that included the striatum were selected. Antigen retrieval (AR) was performed for 20 min at 80 °C (AR buffer: 10 mM Tri-Na citrate buffer, pH 9 + 0.05% Tween 20). Tissue slices were permeabilized for 10 min at room temperature (permeabilization buffer: PBS + 2.5% BSA and 0.2% Triton X-100), followed by blocking for 2 h at room temperature (blocking buffer: PBS + 5% NGS (or NDS) + 1% BSA + 0.1% Triton X-100). Primary antibodies were added at the indicated concentration in blocking buffer and incubated overnight at 4 °C. Secondary antibody was performed for 2 h at room temperature, followed by Hoeschst (1:3,000) for 10 min at room temperature. Tissues were then mounted onto slides and coverslips with Fluoromount-G (Southern Biotech, 0100-01) and stored at 4 °C. For human, 5 µm of paraffin-embedded sections was used. Tissue sections were heated at 65 °C for 30 min and then deparaffinized with 100% CitriSolv (Thermo Fisher Scientific, 04-355-121) for 15 min two times, 100% EtOH for 5 min two times, 95% EtOH for 5 min, 70% EtOH for 5 min, 50% EtOH for 5 min, Milli-Q water for 5 min two times and rehydrated, and then AR was performed with antigen unmaking solution (Vector Laboratories, H-3301) for 20 min at 95 °C. Sections were blocked for 1 h at room temperature with 5% normal goat or donkey serum in 0.1% Triton X-100. Sections were incubated in primary antibody overnight at 4 °C in 1% normal donkey serum in 0.1% Triton X-100. Sections were then incubated in secondary antibodies (1:400 dilution) for 1 h at room temperature in 1× PBS. Secondary antibodies used included Alexa Fluor 488 (1:400; Thermo Fisher Scientific, A-21202) and Alexa Fluor 555 (1:400; Thermo Fisher Scientific, A-31570). Tissues were then treated with TrueBlack Lipofuscin Autofluorescence Quencher (Biotium, 23007) and incubated in Hoeschst for 10 min at room temperature. Sections were then mounted with coverslips using Fluoromount-G.

### Microscopy, IF intensity measurement and co-localization analysis

Images were taken on a Zeiss AiryScan 900 and an Olympus FluoView FV2000 confocal system, with ×40 and ×63 objectives. Images were processed using AiryScan software. Images were taken with the same acquisition settings. Images were then imported to Imaris imaging software version 9 for post-imaging analysis. AL body images had background subtraction to make the phenotype clearer; however, no intensity measurements or statistics were performed. For image analysis, images had all ‘auto-adjustment’ settings reset to raw values. Next, the exact brightness and contrast, minimum/maximum and gamma (default value of 1) were applied to all images for comparison and analysis. For intensity measurement, each cell containing nuclear IF signal was quantified with the Imaris surface tool (version 10) and CellProfiler (version 4.2.6). Normalization was performed between animals by dividing by surface volume. Statistical analysis was performed with unpaired two-tailed *t*-test between HD versus NT and one-way ANOVA with multiple comparisons where appropriate.

### RNA library preparation and sequencing

Starting from mouse frozen cortex, striatal tissue or iPSCs, 1 µg of RNA was extracted and DNase treated using TRIzol (Invitogen) and the manufacturerʼs procedure. The isolated RNA was then used for paired-end library preparation with Illumina TruSeq with Ribo-Zero and sequenced on an Illumina NovaSeq 6000 using the S4 flowcell. RNA quality and concentration were determined with a Bioanalyzer and quantified with Qubit. Targeted read depth was 50 million reads per sample using paired-end sequencing, 100 base pairs (bp). FASTQ files were subjected to FASTQC (version 0.11.7) for quality control analysis, aligned to mm10 or hg38 using STAR aligner (version 2.7.0)^101^. Reference genomes were as follows: human, Homo_sapiens.GRCh38.104.gtf and Homo_sapiens.GRCh38.dna.primary_assembly.fa; mouse, gencode.vM25.annotation.gtf and GRCm38.p6.genome.fa. A count matrix was generated using featureCounts (version 2.0.1)^102^; differential gene analysis was performed with DESeq2 (version 1.42.0)^103^; and genes with low expression were filtered out. For plotting of normalized counts, counts were normalized with trimmed mean of M-values (TMM) in the edgeR package (version 4.0.16)^104,105^. GO analysis and statistics were performed using GOrilla^106^. Motif enrichment was performed with HOMER (http://homer.ucsd.edu/) (version 4.11.1), and plotting was performed with ggplot2 (https://ggplot2.tidyverse.org/) (version 3.4.1).

### AS and CE analysis

rMATS^60^ was used for analysis of AS sites from RNA-seq data. The following options were used: [–od ‘nt_vs_hd’–tmp ‘tmpFolder’ -t paired–readLength 101–cstat 0.0001–libType fr-firststrand–nthread 20 –novelSS]. For novel splice sites, rMATS was re-run with the option –NovelSS. For analysis of AS of single-nuclei RNA-seq data, DESJ-detection was used according to Liu et al.^66^. In every instance, only splice sites with FDR < 0.05 were considered significant. MAJIQ (2.4.dev3 + g85d0781) and LeafCutter (version 0.2.9) analysis were performed following Brown et al.^34^ and Ma et al.^35^.

### RASL-seq

HD R6/2 (*n* = 3 NT, 3 R6/2), Q150 (*n* = 5 WT, 4 HET, 3 HOMO) and Q175 (*n* = 5 WT, 4 HET, 4 HOMO) mouse models at 3 months, 7 months and 18 months, respectively, were used to construct libraries. In total, 9,496 primer pairs targeting known splice junctions were used to amplify libraries for the Illumina sequencing platform. Standard analyses were performed as outlined by Li et al.^98^. In brief, total RNA was mixed with 2× binding cocktail: 40 mM Tris-HCl, pH 7.6, 1 M NaCl, 2 mM EDTA, 0.2% SDS, 5 nM each oligo probe, 100 nM biotinylated oligo(dT)25 and 5 μl of streptavidin-coated magnetic beads. Samples were then heated at 65 °C to denature RNA, followed by 45 °C for 1 h for probe annealing. Next, samples were washed 5× with 1× ligation buffer (T4 DNA ligase (Fermentas, EL0011)). Ligation was carried out at 37 °C for 1 h with 5 U of T4 DNA ligase. Samples were then washed 3× and resuspended in 30 µl of water. Next, samples were incubated at 65 °C for 5 min to release ligated probes. Then, 5 µl of ligated probes was mixed with 10 µl of PCR cocktail containing 1× AmpliTaq GOLD PCR buffer (Life Technologies, N8080241), 0.6 μM P5 primer, 0.6 μM barcoded primer, 1.5 mM MgCl_2_, 200 μM dNTPs and 0.45 U AmpliTaq Gold DNA polymerase, followed by the PCR: (1) 94 °C for 10 min; (2) 20–25 cycles at 94 °C for 30 s; (3) 58 °C for 30 s; and (4) 72 °C for 30 s. The amount of pooled and purified PCR products was quantified with a Qubit fluorometer before sequencing. Raw data can be found in Supplementary Data 11 and 12.

### PacBio Iso-seq

Ten micrograms of TRIzol-extracted RNA from mouse cortex and striatum was treated with Terminator 5′-Phosphate-Dependent Exonuclease (Lucigen, TER51020) according to the manufacturer’s protocol. RNA was purified using a Zymo RNA Clean & Concentrator-5. Spike-in RNA control (0.3 µl), SIRV set 3 (Lexogen) diluted 1:10, was added to 300 ng of purified RNA before cDNA synthesis. A modified protocol was used for cDNA synthesis using SuperScript IV and template-switching oligos^107^. cDNA was pre-amplified using KAPA HiFi HotStart Ready Mix (Roche) for 27 cycles. Iso-seq libraries were prepared using the SMRTbell Template Express Prep Kit 2.0 (PacBio) using 300 ng of cDNA input per sample according to the manufacturer’s protocol. After library preparation, DNA was purified with 0.45× AMPure PB beads, washed with 80% EtOH twice and eluted with 100 µl of elution buffer. DNA was eluted from the beads at 37 °C for 15 min. A second round of purification with AMPure PB beads was included, and each DNA library was eluted in 10 µl. DNA library quality and consistency were verified with a Qubit and a Bioanalyzer and loaded onto the PacBio Sequel II sequencer. PacBio sequencing data were analyzed using SMRT Analysis software (version 9.0) (PacBio). Consensus reads were generated using ccs 4.2.0 with options –skip-polish–minLength 10–minPasses 3–min-rq 0.9–min-snr 2.5. Adapters were removed with lima 1.11.0 with options –isoseq–min-score 0–min-end-score 0–min-signal-increase 10–min-score-lead 0. Full-length non-chimeric reads were extracted, were oriented to the correct strand and had polyA tails clipped using isoseq3 refine 3.3.0 with options –min-polya-length 20–require-polya. The resulting full-length non-chimeric reads were mapped to mm10 reference genome using minimap2 (2.17-r941)^108^ with options -ax splice:hq -uf -MD. TranscriptClean (version 2.0.2) (https://github.com/mortazavilab/TranscriptClean) was used for error correction with the option –canonOnly, and TALON (version 5.0)^64^ was used for error correction and transcript identification and quantification. The module talon_label_reads with option –ar 20 was used to compute the fraction of As at the ends of read alignments. TALON databases of mouse (Ensembl 87 annotations) were created using talon_initialize_database with options –l 0–5p 500–3p 300. The TALON module was run with default parameters to identify transcripts using the initialized database. Filtered transcript lists, which are limited to known and consistently observed transcripts, were generated using talon_filter_transcripts. Filtered and unfiltered transcript abundances were obtained using the talon_abundance module.

### TDP-43 and m6A eCLIP-seq

eCLIP-seq was carried out with commercial kits from ECLIPSEBIO with the data analysis add-on (https://eclipsebio.com). Male and female R6/2 and corresponding NT animals from the matching cohort (*n* = 4 per group) were aged to 12 weeks. Striatum and cortex were dissected and flash frozen in liquid nitrogen. For m6A eCLIP, total RNA was extracted using TRIzol. mRNA was enriched using poly-dT beads (New England Biolabs) and cleaned with Zymo Clean & Concentrator-5 according to standard protocol and then followed the ECLIPSEBIO protocol. For TDP-43 eCLIP, samples were homogenized to a powder with a BioSpec 59012N Tissue Pulverizer. Samples were next UV crosslinked 2× at 400 mJ cm^−2^. Samples were collected in a 1.5-ml tube. Next, the RBP-eCLIP kit from ECLIPSEBIO with data analysis was used following RBP-eCLIP Protocol V2.01R to generate TDP-43 eCLIP-seq libraries (based on Van Nostrand et al.^79^). Then, 2 µg of TDP-43 antibody was used for each IP (Bethyl Laboratories, A303-223A). IP and corresponding input samples were sequenced at 50 million reads using 100-bp paired-end sequencing on the NovaSeq 6000 with the S4 flow cell. Male and female samples were compared using irreproducibility discovery rate (IDR) incorporated into CLIPper peak finder^109^ to identify reproducible binding peaks and filtered by *P* < 0.001 and log(FC) > 3. A density plot was created using IDR peak location and IP BAM files with deepTools (version 3.5.1)^110^. A metagene plot was created with MetaPlotR (version 2.0.5)^111^ using the following standard pipeline^111^.

### LC–MS analysis of m6A

Processing of RNA for LC–MS-based m6A analysis was performed as described in Mathur et al.^112^. In brief, 100 ng of twice-purified polyA-RNA was digested with 1 U of nuclease P1 (Sigma-Aldrich, N8630) for 2 h at 37 °C, followed by treatment of 1 U of alkaline phosphatase (Sigma-Aldrich, P5931) for 2 h at 37 °C. Then, 21.5 μl of the purified nucleoside sample (equivalent of 25 ng of RNA) was mixed with 2× volume (43 μl) of acetonitrile. Samples were centrifugated at 16,000*g* for 10 min at 4 °C, and 40 µl of supernatant was loaded into MS vials. Adenosine and m6A signals were analyzed by a quadrupole Orbitrap mass spectrometer (Thermo Fisher Scientific) coupled to hydrophilic interaction chromatography (HILIC) via electrospray ionization. LC separation was performed on an Xbridge BEH amide column (2.1 mm × 150 mm, 2.5-µm particle size, 130-Å pore size; Waters) at 25 °C using a gradient of solvent A (5% acetonitrile in water with 20 mM ammonium acetate and 20 mM ammonium hydroxide) and solvent B (100% acetonitrile). The flow rate was 200 µl min^−1^. The LC gradient was as follows: 0 min, 75% B; 3 min, 75% B; 4 min, 50% B; 7 min, 50% B; 7.5 min, 75% B; 11 min, 75% B. The autosampler temperature was set at 4 °C, and the injection volume of the sample was 3 μl. MS data were acquired in positive ion mode with a full-scan mode from *m*/*z* 240 to 290 with 140,000 resolutions. Data were analyzed using El-MAVEN software (version 0.12.0). Adenosine (Sigma-Aldrich, A9251) and m6A (Selleckchem, S3190) standards were quantified based on standard calibration curves using authentic standards.

### Figure artwork

Figures 1a and 3d and Supplementary Fig. 4a contain artwork created in BioRender (Thompson Lab, https://biorender.com/d90p843 (2024)).

### Reporting summary

Further information on research design is available in the Nature Portfolio Reporting Summary linked to this article.

## Online content

Any methods, additional references, Nature Portfolio reporting summaries, source data, extended data, supplementary information, acknowledgements, peer review information; details of author contributions and competing interests; and statements of data and code availability are available at 10.1038/s41593-024-01850-w.

## Supplementary information

Supplementary Information Supplementary Figs. 1–11, figure legends for Supplementary Data 1–12 and supplementary figures and references.

Reporting Summary

Supplementary Data 1–12 Supplementary Data legends: Supplementary Data 1: Column A, list of RBPs that are enriched in the HDinHD proteome dataset; column C, list of RBPs predicted from SONAR^57^. Supplementary Data 2: DGE data from DESeq2 (ref. ^103^). *n* = 10 (five males, five females) per genotype were used for analysis. Statistical *P* values listed were determined using the DESeq2 package. Left side shows cortex data; right side shows striatum data. Filtered for events with adjusted *P* < 0.05. Supplementary Data 3: List of CHDI’s 266 striatal genes that are dysregulated in HD mouse models. Column C shows direction of change relative to HD condition. Supplementary Data 4: List of significant (FDR < 0.05) SEs as annotated by rMATS. Cortex, rows 3–3,524; striatum, rows 3,529–7,689. Human (Al-Dalahmah et al., Human SE), rows 7,691–12,372. Human (Labadorf et al., Human SE), rows 12,377–17,821. *P* values were determined and output by rMATS. Supplementary Data 5: Output from MAJIQ and LeafCutter. Dataset was filtered for probability changing >.90 for MAJIQ and deltaPSI >.10 or −.10. MAJIQ cortex, rows 3–203. MAJIQ striatum, rows 207–443. LeafCutter cortex, rows 447–610. LeafCutter striatum, rows 614–857. Supplementary Data 6: DGE data from DESeq2 (ref. ^103^). DESeq2 output from HTT (18Q) versus mHTT (50Q) day 37 MSNs. *n* = 3 per genotype were used for analysis. Statistical *P* values listed were determined using the DESeq2 package. Filtered for events with adjusted *P* < 0.05. Supplementary Data 7: DGE data from DESeq2 (ref. ^103^). DESeq2 output from HTT (18Q) non-targeting siRNA versus TDP-43 KD siRNA at day 37 MSNs. *n* = 3 per genotype were used for analysis. Statistical *P* values listed were determined using the DESeq2 package. Filtered for events with adjusted *P* < 0.05. Supplementary Data 8: List of DEGs that overlapped between TDP-43 KD and mHTT dependent (*P* < 0.05) as determined by the hypergeometric test. Column A contains genes that overlap with upregulated genes in the mHTT condition; column B contains genes that overlap with downregulated genes in the mHTT condition. Supplementary Data 9: Summary of human postmortem brain tissues used in this study. F, female; HD-X, with X indicating Vonsattel grading; M, male; PMD, postmortem delay. Supplementary Data 10: List of antibodies used in this study. Supplementary Data 11: List of primer pairs used for RASL-seq. Supplementary Data 12: RASL-seq raw data for R6/2 (starting at row 1), Q150 (starting at row 1,748) and Q175 (starting at row 3,538).

## Data Availability

Public datasets used in this study included Polymenidou et al. (Gene Expression Omnibus (GEO): GSE27394)^23^; Al-Dalahmah et al. (requested from author)^63^; Labadorf et al. (GEO: GSE64810)^78^; and Šušnjar et al. (GEO: GSE171714)^73^. All sequencing raw data generated in this study are deposited in the GEO or provided in Supplementary Information, with the following accession numbers: GSE278354, GSE278893, GSE279460 and GSE281847. All data are available in the main text or Supplementary Information; all other data will be made available upon reasonable request.
