# Supplementary material for: Aberrant splicing in Huntington’s disease accompanies disrupted TDP-43 activity and altered m6A RNA modification
Source: Nat Neurosci. 2025 Jan 6;28(2):280–92. doi: 10.1038/s41593-024-01850-w (PMC11802453; doi:10.1038/s41593-024-01850-w)
Supplement: Supplementary file 1 — Supplementary Figs. 1–11, figure legends for Supplementary Data 1–12 and supplementary figures and references. [file 41593_2024_1850_MOESM1_ESM.pdf]

nature neuroscience

Supplementary information

https://doi.org/10.1038/s41593-024-01850-w

Aberrant splicing in Huntington's disease accompanies disrupted TDP-43 activity and altered m6A RNA modification

In the format provided by the authors and unedited

Supplementary Figures:

Supplementary Figure 1

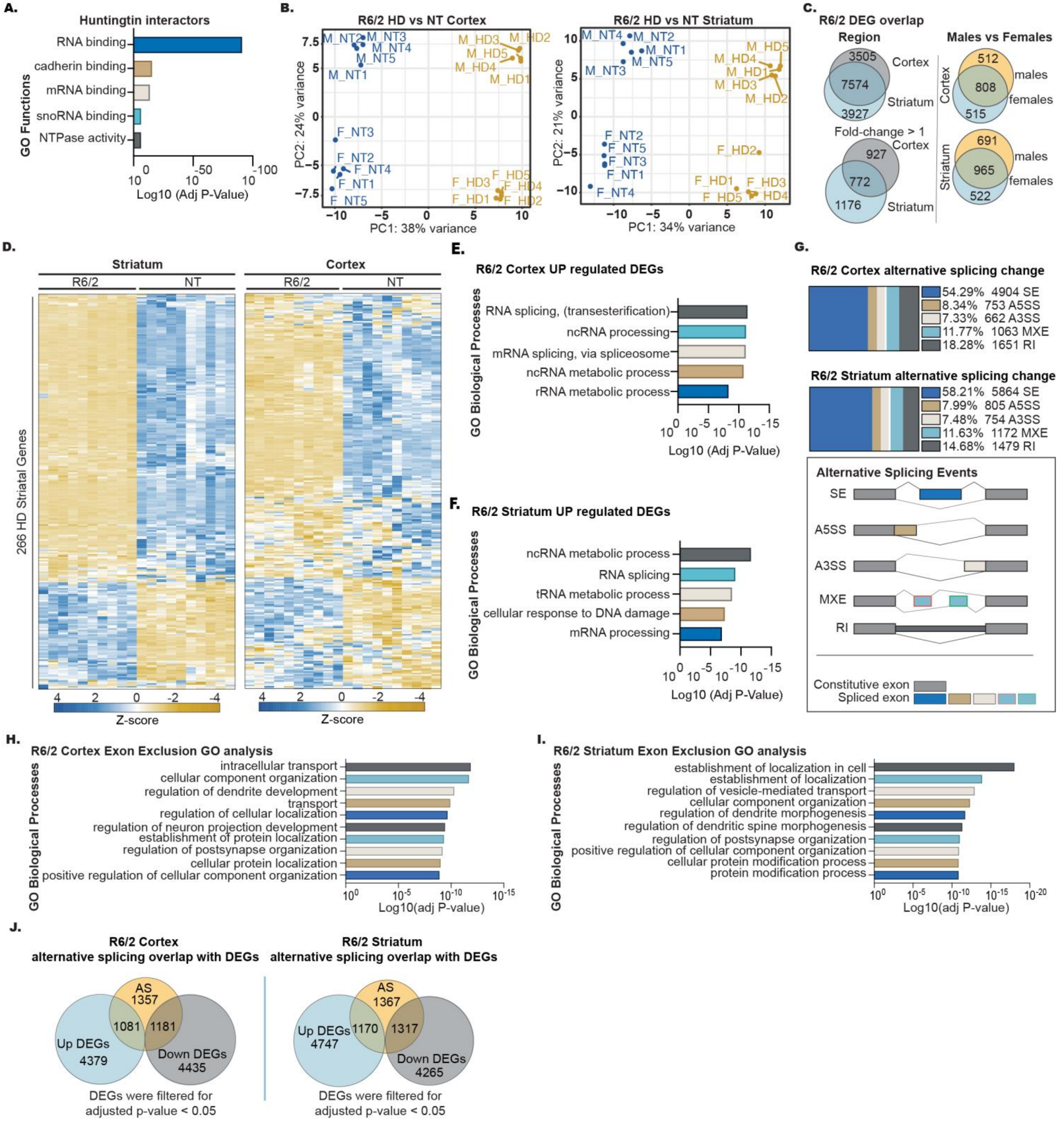

Supplementary Figure 1. Alternative splicing changes in HD mouse models.

(A) Gene ontology analysis [1] for molecular functions of 4182 predicted protein interactors of Huntingtin. p-value was dervied within the GO-rilla analysis package [2]. X-axis represents the log10(adjusted p-value) derived from statistical testing within the GO-rilla analysis package. (B) Principal Component Analysis (PCA) from the RNA-seq data from striatum and cortex of 12 weeks old R6/2 mice. Separation between groups is observed on PC1 by genotype and on PC2 by sex. Count data was generated with FeatureCount [3], blue are NT samples and yellow are R6/2 samples. F = female / M = male. (C) Venn diagram showing overlap of differentially expressed genes (DEGs) output from DESeq2 [4] between striatum, cortex, male, and female samples. Significance was determined by adjusted p-value<0.05, male vs female DEGs are filtered for log2foldchange>1. (D) Heatmap showing gene expression changes in RNA-seq data of the 266 striatal DEGs defining the signature reported by Obenauer et al. [5]. Heatmap contains 10 males and 10 female samples with 5 NT and 5 HD from each group. Gene ontology analysis for biological processes of upregulated genes identified by DEseq2 [4] in 3 months old R6/2 mice from cortex (E) and (F) striatum. Gradient scale represents Z-scores of normalized gene counts. (G) Significant splicing event changes in the cortex (top) and striatum (bottom) from rMATs [6] analysis (FDR < 0.05). Gene Ontology analysis for biological process of the significant skipped exons in the R6/2 cortex (H) and striatum (I). (J) Overlap between significant alternatively splicing events from rMATs and genes up and downregulated in the R6/2 cortex (left) and striatum (right). DEGs were filtered for adjusted p-value < 0.05. p-values for C, E, F, H, I, & J were derived from statistical testing within the DESeq2 analysis package [4].

Supplementary Figure 2

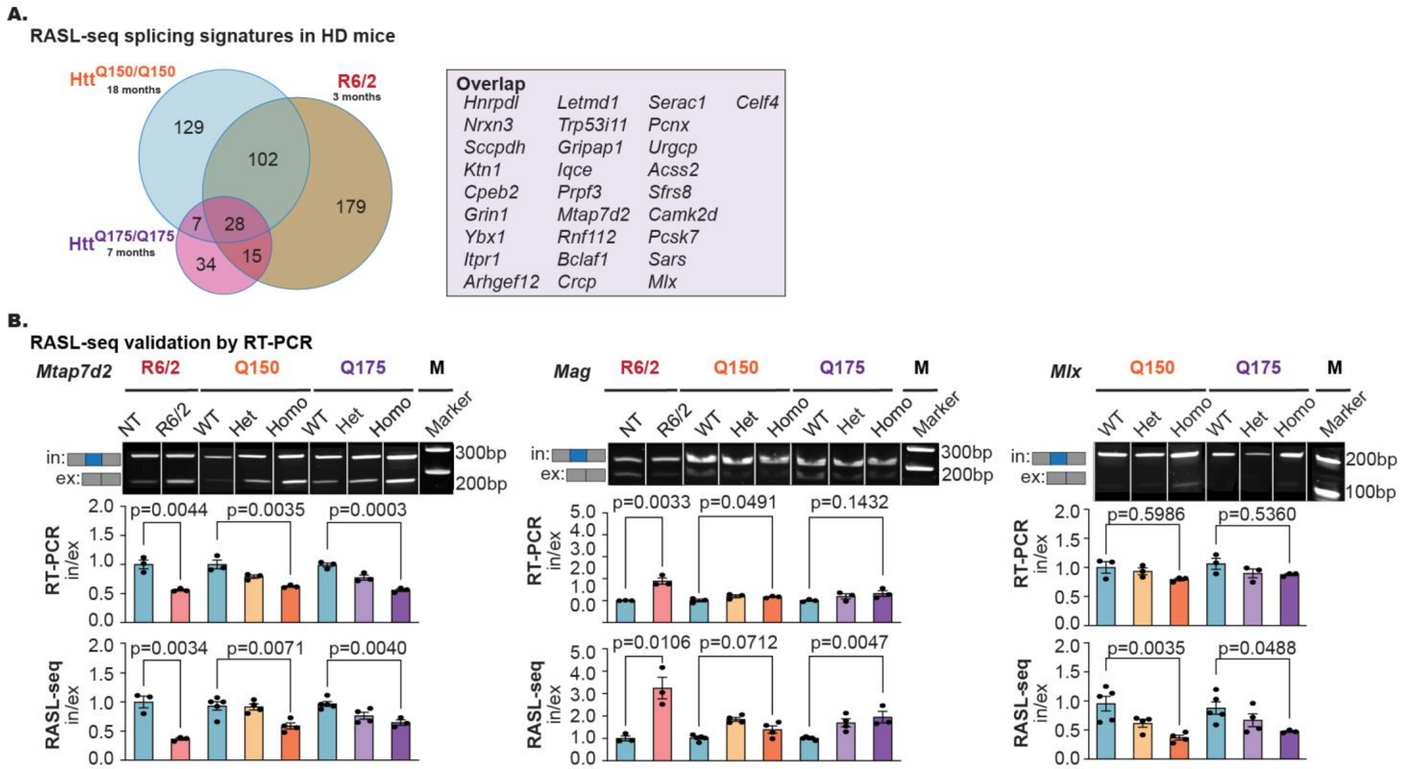

Supplementary Figure 2. RASL-seq validation.
(A) Venn diagram showing the overlap of significant splicing changes identified by RASL-seq [7] between the R6/2 (R62=3, NT=3), Q150 (WT=5, Het=4, Homo=4) and Q175 mice(WT=5, Het=4, Homo=3). (B) PCR primers were generated to validate (RT-PCR, n=3 per genotype) the included and excluded isoforms detected by RASL-seq. Statistical significance was determined by a one-way ANOVA with Tukey's multiple comparison test for Q150 and Q175, and by unpaired two-tailed t-test for R6/2. Mtap7d2: R6/2: RT-PCR, p-value=0.0044, t=5.789, df=4, F=22.25, 95%CI=-0.6518 to -0.2292 | RASL-seq, p-value=0.0034, t=6.227, df=4, F=24.30, 95%CI=-0.9215 to -0.3532 || Q150: RT-PCR, p=0.0035, df=6, F=15.53, 95%CI= 0.1696 to 0.5871 | RASL-seq, p=0.0071, df=10, F=9.273, 95%CI=0.1044 to 0.5834 || Q175, RT-PCR, p=0.0003, df=6, F=39.14, 95%CI=0.2811 to 0.5795 | RASL-seq, p=0.0040, df=9, F=10.96, 95%CI=0.1205 to 0.5219 || Mag: R6/2: RT-PCR, p-value=0.00334, t=6.292, df=4, F=911.3, 95%CI=0.4957 to 1.279| RASL-seq, p-value=0.0106, t=4.530, df=4, F=20.06, 95%CI=0.8693 to 3.623 || Q150: RT-PCR, p=0.0491, df=6, F=3.451, 95%CI=-0.4058 to 0.07928 | RASL-seq, p=0.0712, df=10, F=16.04, 95%CI=-0.7734 to 0.03191 || Q175, RT-PCR, p= 0.1432, df=6, F=2.525, 95%CI=-0.7645 to 0.1201| RASL-seq, p= 0.0047, df=9, F= 11.16, 95%CI=-1.549 to -0.3390|| Mlx: Q150: RT-PCR, p=0.3845, df=6, F=1.126, 95%CI=-0.4502 to 0.2274| RASL-seq, p=0.0044, df=10, F=9.846, 95%CI=0.2189 to 0.9472|| Q175, RT-PCR, p=0.5360, df=6, F=0.9074, 95%CI=-0.2222 to 0.4786| RASL-seq, p=0.0488, df=9, F=4.030, 95%CI=0.002418 to 0.8346||. Data are presented as mean values +/- SEM.

Supplementary Figure 3

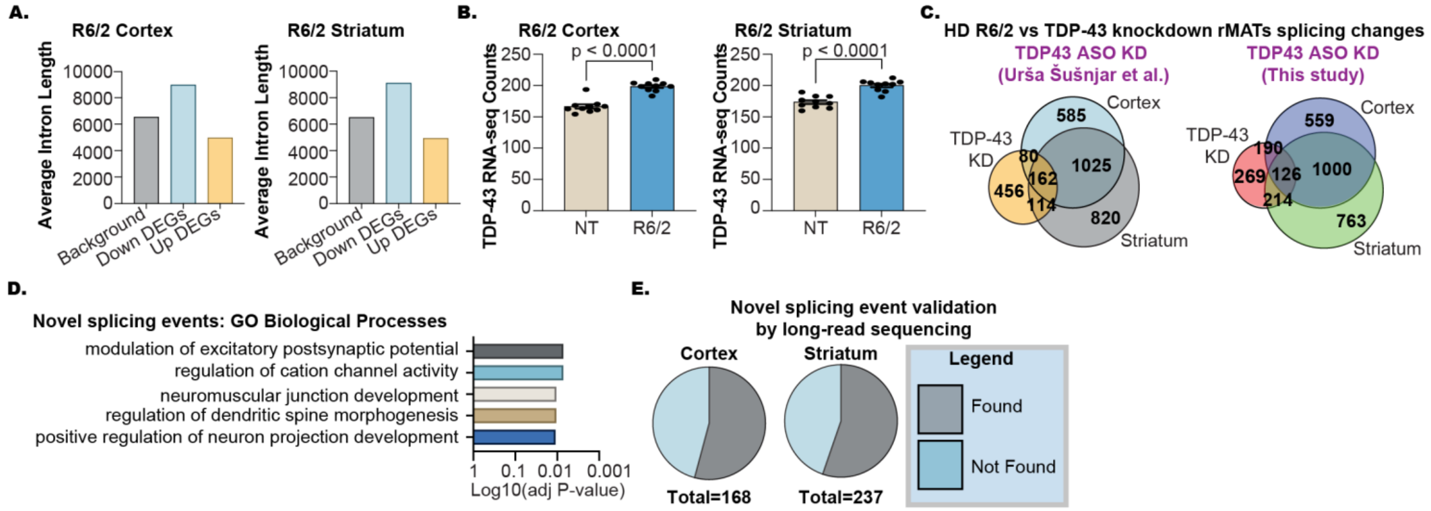

Supplementary Figure 3. Transcriptomic similarities between TDP-43 loss-of-function and HD R6/2. (A) For all expressed genes (base mean > 1, from DeSeq2) the intron length was pulled using the mm10 mouse genome annotation and the average intron length per group was calculated (all introns / sum of introns). Intron length in genes differentially expressed in R6/2 mice compared to controls (Up DEGs and Down DEGs) was compared to intron length in non-differentially expressed genes (Background). (B) Normalized RNA-seq counts for TDP-43 mRNA in cortex from 12-week-old R6/2 mice generated from FeatureCount. *n=10* biological replicates per genotype (5 males, 5 females) Significance determined by unpaired two-tailed student t-test (cortex: p-value < 0.0001, t=7.749, df=18, F=2.032, 95%CI=23.22-40.50 | striatum: p-value < 0.0001, t=6.402, df=18, F=1.129, 95%CI=17.74-35.07). Data are presented as mean values +/- SEM. (C) Comparison of R/2 versus NT rMATs [6] SE output and TDP-43-dependent rMATs SE output (Šušnjar, 2022 [8]) in mouse neurons and whole cortex and striatal tissues (*n=5*/treatment). (D) Gene Ontology analysis of mHTT dependent novel splicing events for biological processes. (E) PacBio Iso-Seq validation of Novel splicing events, isoforms were searched for the inclusion or exclusion of novel splice products.

Supplementary Figure 4

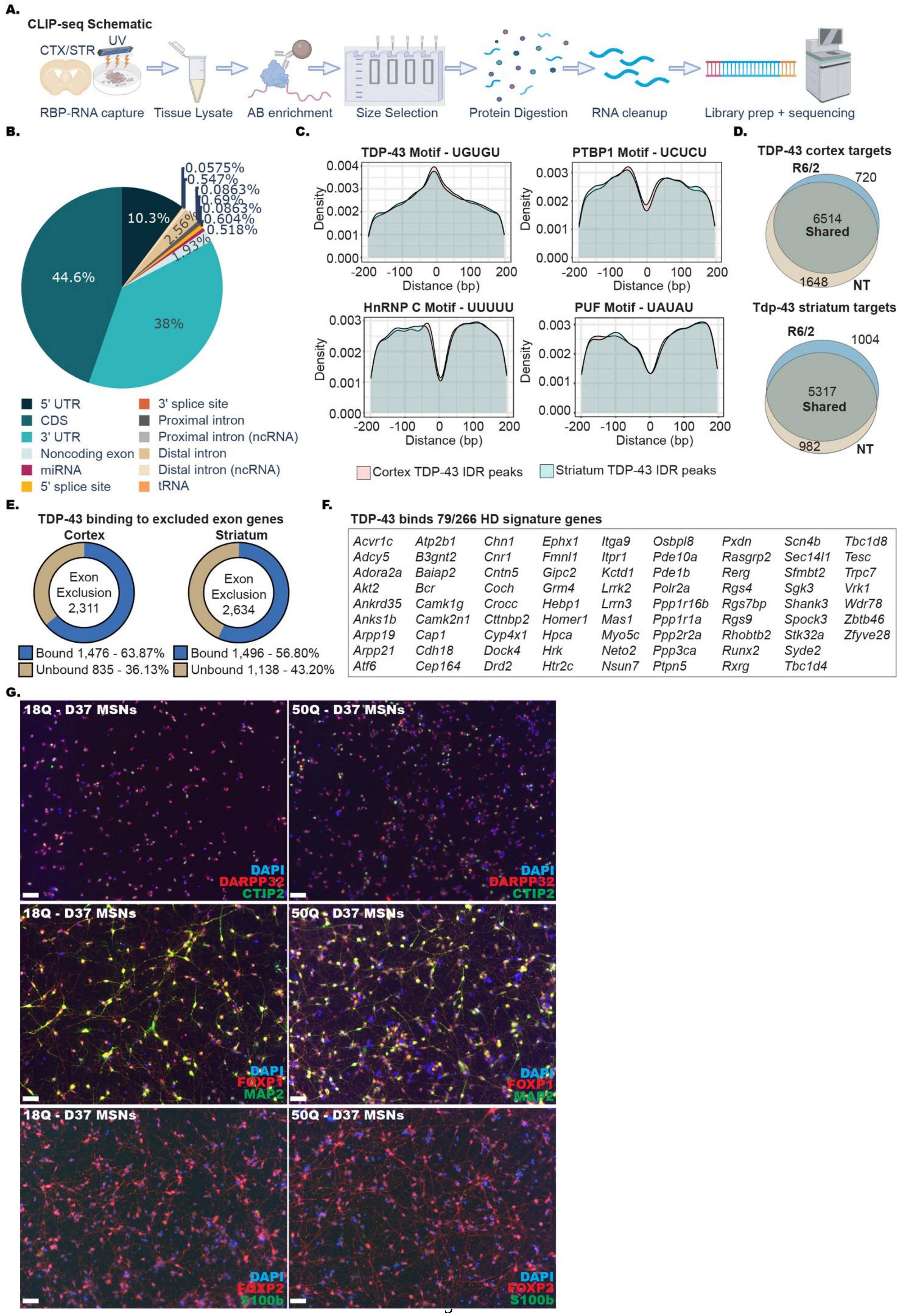

Supplementary Figure 4. TDP-43 binds to HD R6/2 dysregulated genes.

(A) Schematic of TDP-43 eCLIP-seq experiment. (B) Pie chart showing the classification of TDP-43 peaks in the genome. (C) TDP-43 eCLIP-seq IDR peaks centered at the 0 position. Y-axis represents the density at which the UGUGU, UCUCU, UUUUU, and UAUAU motifs can be found. (D) Venn diagram showing the number of genes that overlaps between R6/2 vs NT TDP-43 CLIP peaks in the cortex and striatum. (E) TDP-43 peaks overlapping with HD R6/2 exon exclusion (CTX: 1,476/2,311, STR: 1,496/2,634) (1,496/2,634). (F) List of 79/266 HD R6/2 striatal signature genes that are bound by TDP-43 from TDP-43 CLIP-seq reanalysis. (G) Representative QC staining for d37 MSNs derived from iPSCs. Scale bar = 50 μm. 3 images were taken per stain per replicate with similar results represented above.

Supplementary Figure 5

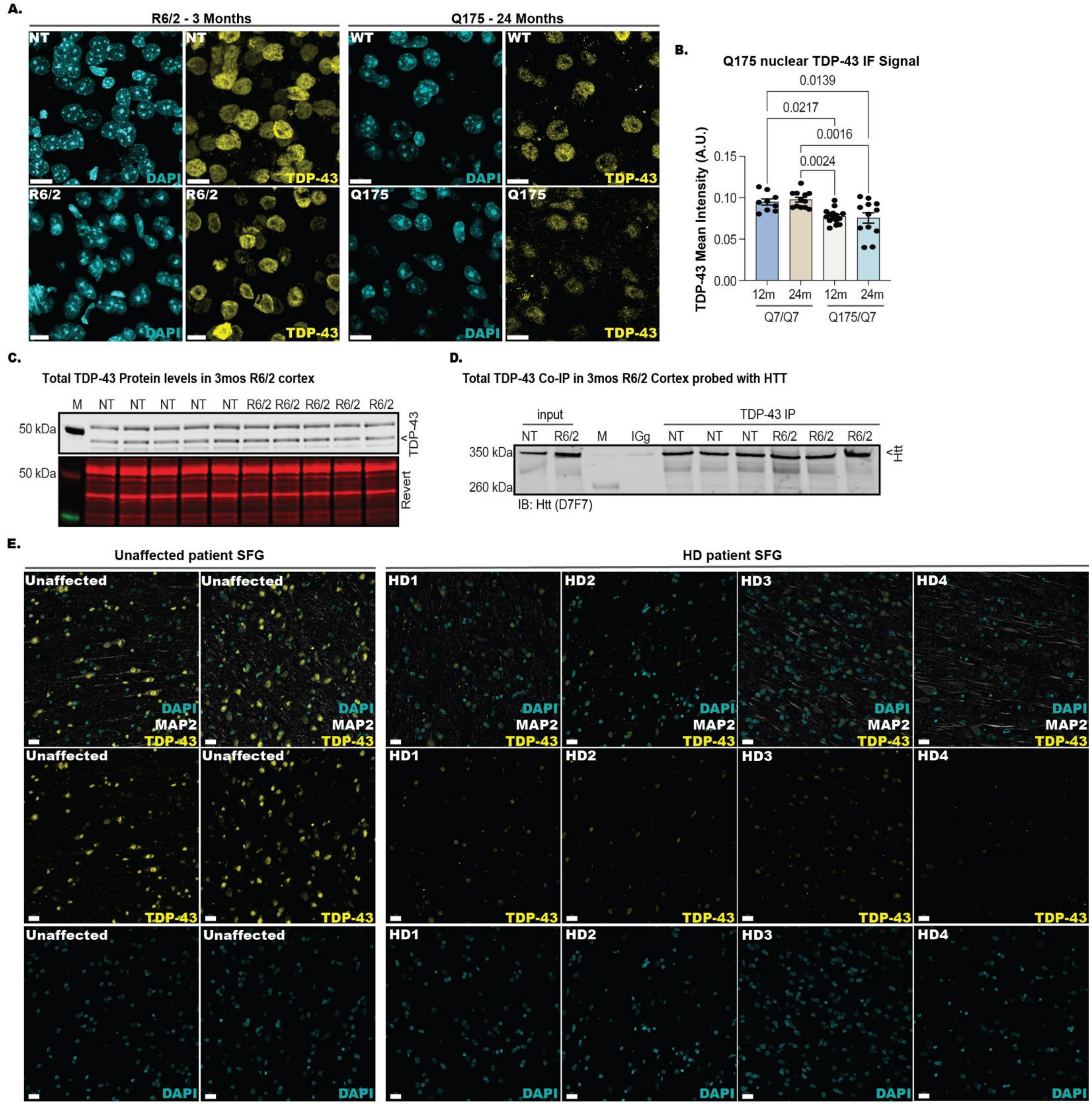

Supplementary Figure 5. Altered TDP-43 nuclear protein expression in HD systems.

(A) Immunofluorescence (IF) staining of the cortex of 3-month-old HD R6/2 mice, 24-month-old Homozygous Q175 mice, and wild-type control littermates (NT or WT). Scale bar = 10 μm. Cyan is the nuclei marker DAPI, yellow shows total TDP-43 staining. (B) IF Quantitation of TDP-43 nuclear signal by CellProfiler from 12-month-old and 24-month-old Q7/Q7 and Q175/Q7. (Q7/Q7 12m :*n=3* | Q7/Q7 24m: *n=4* | Q175/Q7 12m: *n=5* | Q175/Q7 24m: *n=4*). Each datapoint represents the average TDP-43 nuclear intensity from one imaging region (40X) of the cortex (3 regions per animal) within a genotype. Data are presented as mean values +/- SEM. Statistical significance determined by one-way ANOVA with Tukey's multiple comparisions test. Only significant p-values/comparisons are shown. (C) Western blot by Li-COR using 5ug of lysate per lane from cortex of 3-month-old NT and HD R6/2 males. Red staining shows REVERT total protein staining for loading control. Arrow indicates expected TDP-43 band. (D) Co-immunoprecipitation using a TDP-43 antibody followed by Western blot analysis with an HTT antibody using lysates from age matched (3-month-old) NT control or HD R6/2 animals. M=marker, IGg= Igg negative control. C, and D were repeated at least 3 times with similar results represented above. (E) Representative IF staining images of superior frontal gyrus from HD patients compared to non-HD control individuals showing decreased TDP-43 (yellow) signal intensity. Scale bar = 20 μm.

Supplementary Figure 6

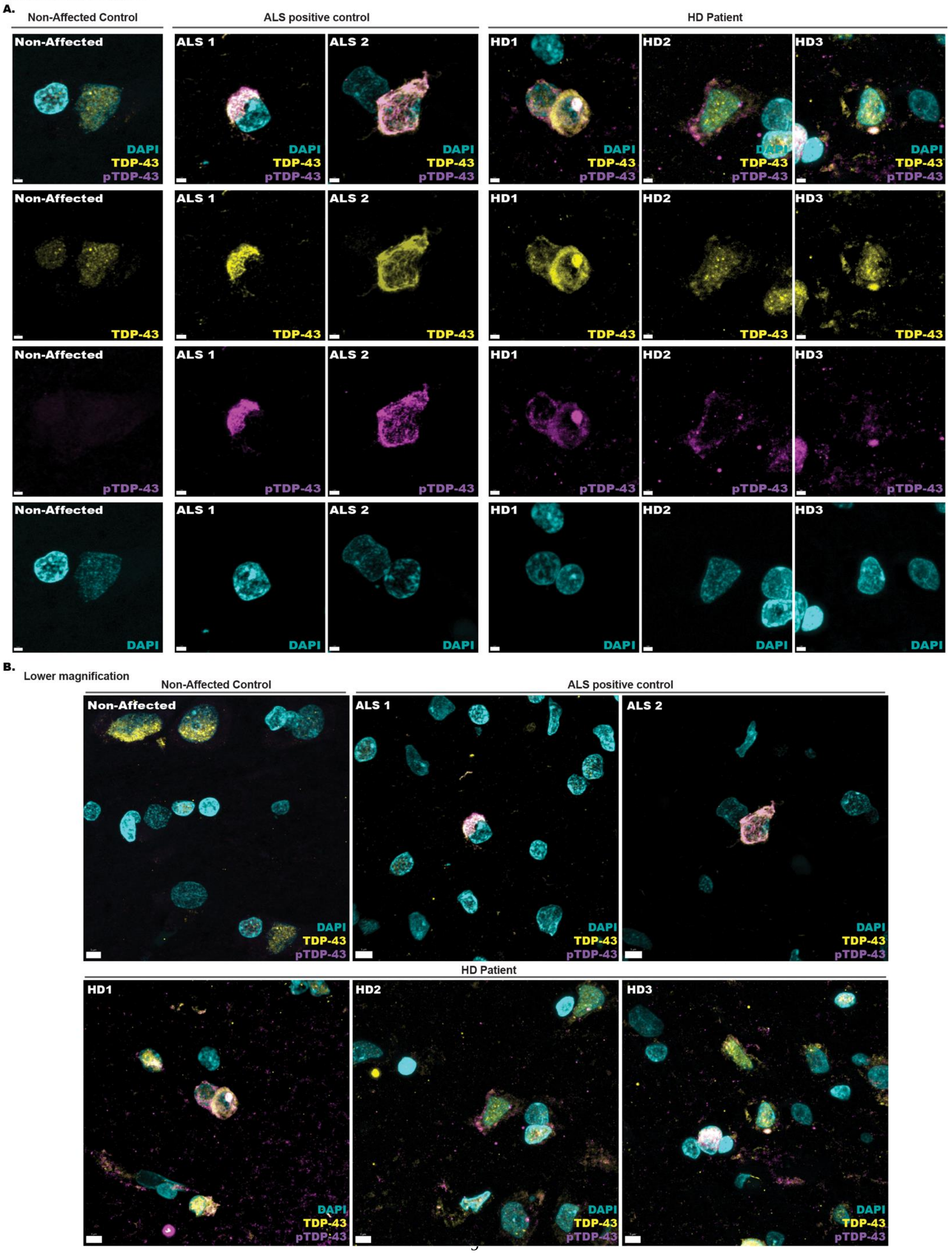

Supplementary Figure 6. TDP-43 protein mislocalization in HD patients.
(A) Representative high magnification IF staining images in the motor cortex from an ALS patient (positive control), Non-affected control patients (negative control), compared to the superior frontal gyrus (SFG) of HD patients, using antibodies against total TDP-43 (yellow), phosphorylated TDP-43 (purple), and nuclear stain DAPI. Scale bar = 2 μm. (B) Representative low magnification IF staining images in the motor cortex from an ALS patient (positive control) compared to the superior frontal gyrus (SFG) of HD patients, using antibodies against total TDP-43 (yellow), phosphorylated TDP-43 (purple), and nuclear stain DAPI. Scale bar = 5 μm. A, and B were repeated at least 3 times with similar results represented above.

Supplementary Figure 7

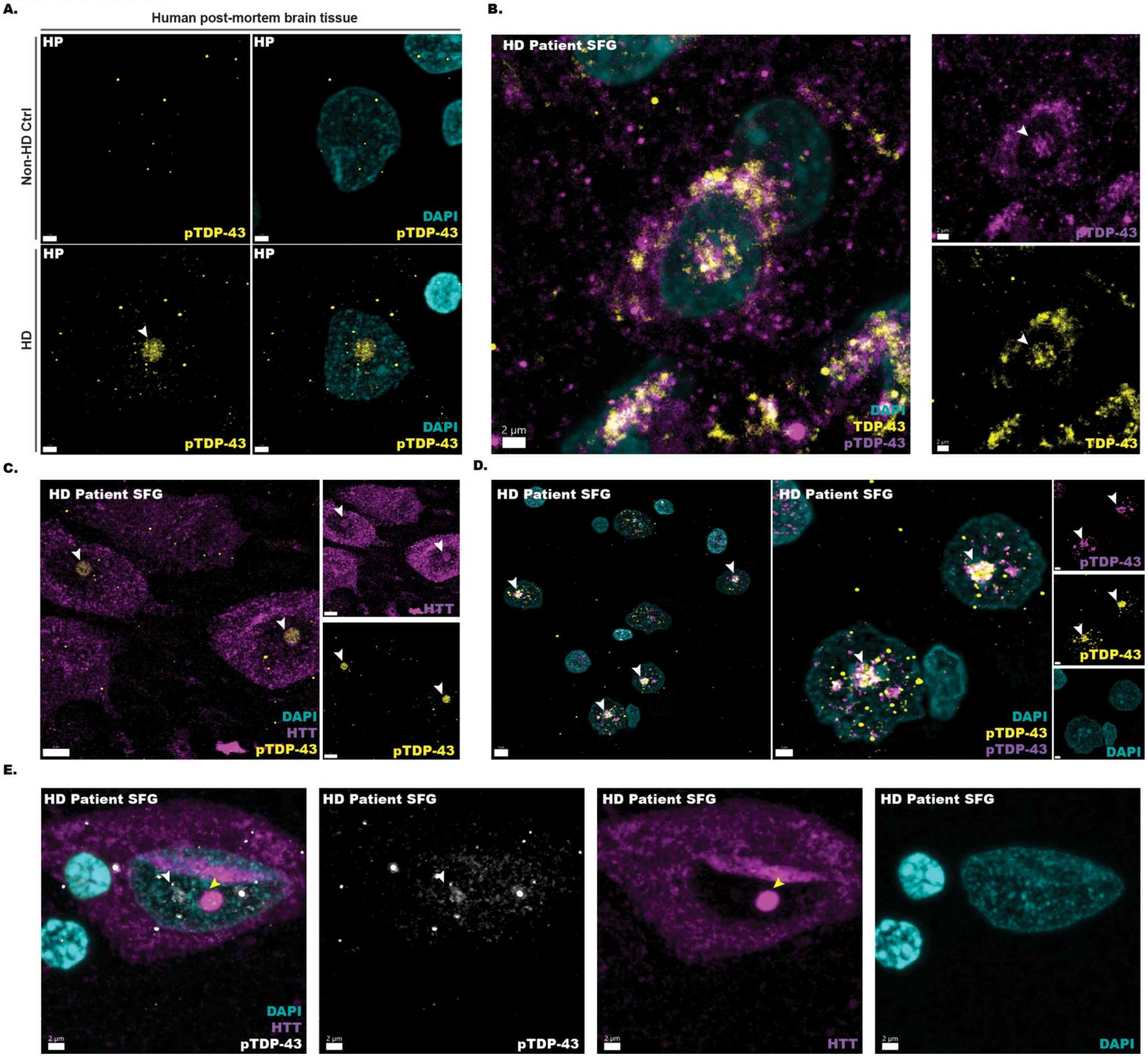

Supplementary Figure 7. TDP-43 AL-bodies in HD patients.

(A) Representative images of immunofluorescence (IF) staining of pTDP-43 (yellow) nuclear AL-bodies identified in Hippocampal regions of HD patients and not in unaffected control individuals. White arrow indicates AL-bodies. Scale bar = 2 μm. (B) Representative images of immunofluorescence (IF) staining showing co-localization of nuclear pTDP-43 AL-bodies (purple) with total TDP-43 (yellow) in the superior frontal gyrus (SFG) of HD patients. White arrows indicate location of AL-bodies. Scale bar = 2 μm. (C) IF images showing co-localization of nuclear pTDP-43 AL-bodies (yellow) with the HTT antibody 5526 (purple). Scale bar = 5 μm. (D) Lower magnification IF showing the co-localization of two pTDP-43(S409/S410) antibodies: pTDP-43 (purple, Cat#RB3655 (gift from Dr. Leonard Petrucelli) and pTDP-43 (yellow, Cat# Biolegend 50-102-9913). White arrows indicate location of AL-bodies. scalebar=5µm. Higher magnification IF showing the co-localization of two pTDP-43(S409/S410) antibodies: pTDP-43 (purple) and pTDP-43 (yellow). Scale bar = 2 μm. (E) IF images showing co-localization of nuclear pTDP-43 AL-bodies (white) with the HTT antibody 5526 (purple) and the lack of co-localization when canonical nuclear HTT inclusion (yellow arrow) is detected. Scale bar = 2 μm. A, B, C, D, and E were repeated at least 3 times with similar results represented above.

Supplementary Figure 8

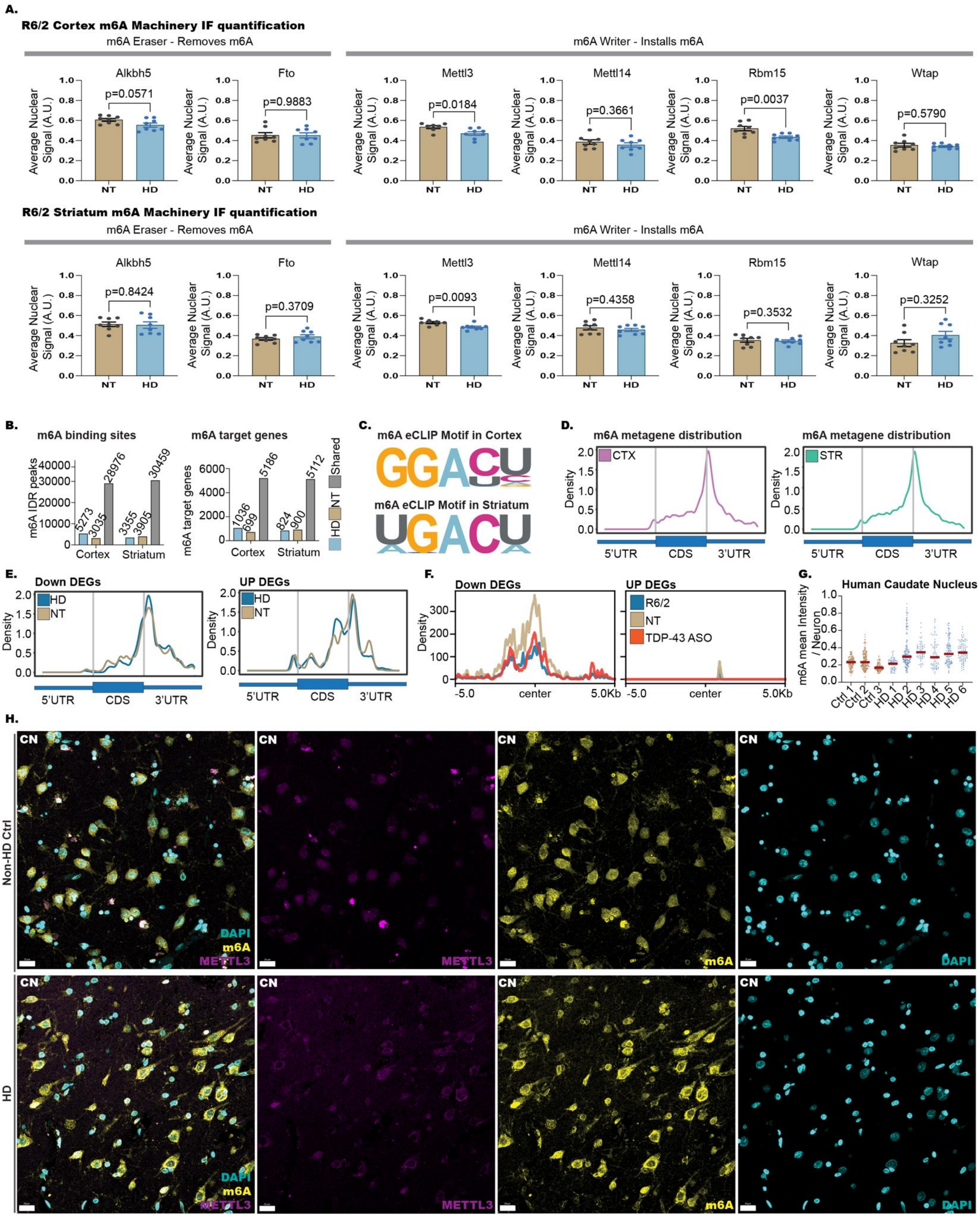

Supplementary Figure 8. m6A machinery proteins in HD systems.

(A) m6A machinery IF quantification by CellProfiler. Statistical significance determined by unpaired student two-tailed t-test. *n=8* (4 males, 4 females) per genotype were quantified each averaging three cortical or striatal regions. Data are presented as mean values +/- SEM. Cortex: Alkbh5: p-value=0.0571, t=2.073, df=14, F=2.177, 95%CI=-0.1054 to 0.001804 | Fto: p-value=0.9883, t=0.01488, df=14, F=1.053, 95%CI=-0.07777 to 0.07670 | Mettl3: p-value=0.0184, t=2.693, df=13, F=1.572, 95%CI=-0.11587 to -0.01270 | Mettl14: p-value=0.3661, t=0.9341, df=14, F=1.079, 95%CI=-0.09563 to 0.03761 | Rbm15: p-value=0.0037, t=3.476, df=14, F=3.235, 95%CI=-0.1389 to -0.03289 | Wtap: p-value=0.5790, t=0.5680, df=14, F=3.882, 95%CI=-0.05754 to 0.03345. Striatum: Alkbh5: p-value=0.8424, t=0.2026, df=14, F=2.087, 95%CI=-0.09154 to 0.07574 | Fto: p-value=0.3709, t=0.9244, df=14, F=2.350, 95%CI=-0.02906 to 0.07307 | Mettl3: p-value=0.0093, t=3.052, df=13, F=1.044, 95%CI=-0.07740 to -0.01324 | Mettl14: p-value=0.4358, t=0.8023, df=14, F=1.805, 95%CI=-0.07727 to 0.03520 | Rbm15: p-value=0.7204, t=0.3653, df=14, F=2.589, 95%CI=-0.05600 to 0.03970 | Wtap: p-value=0.1466, t=1.537, df=14, F=1.234, 95%CI=-0.03143 to 0.1904. (B) Number of m6A-eCLIP sites detected and genes detected. Numbers on top of bars represent counts. (C) Enriched motif for Cortex and Striatum m6A-seq IDR peaks. (D) Metagene plot generated by metaplotR[9] showing the distribution of m6A sites across a metagene. (E) m6A distribution on down and upregulated genes showing altered m6A modifications across transcripts in HD mice. (F) m6A site deposition (localized levels) on Up and Down regulated genes in R6/2 vs NT. (G) Sample separated Dot plot from figure 5H. Each dot represents the average m6A intensity per neuronal cell spanning 5 20X confocal images per patient (3 controls and 6 HD) (H) Lower magnification representative images showing significant increase in m6A IF intensity in HD patient caudate nucleus, Scale bar = 20 μm. H was repeated at least 3 times with similar results represented above.

Supplementary Figure 9

A.

Uncropped blots for Supplementary Figure 5C

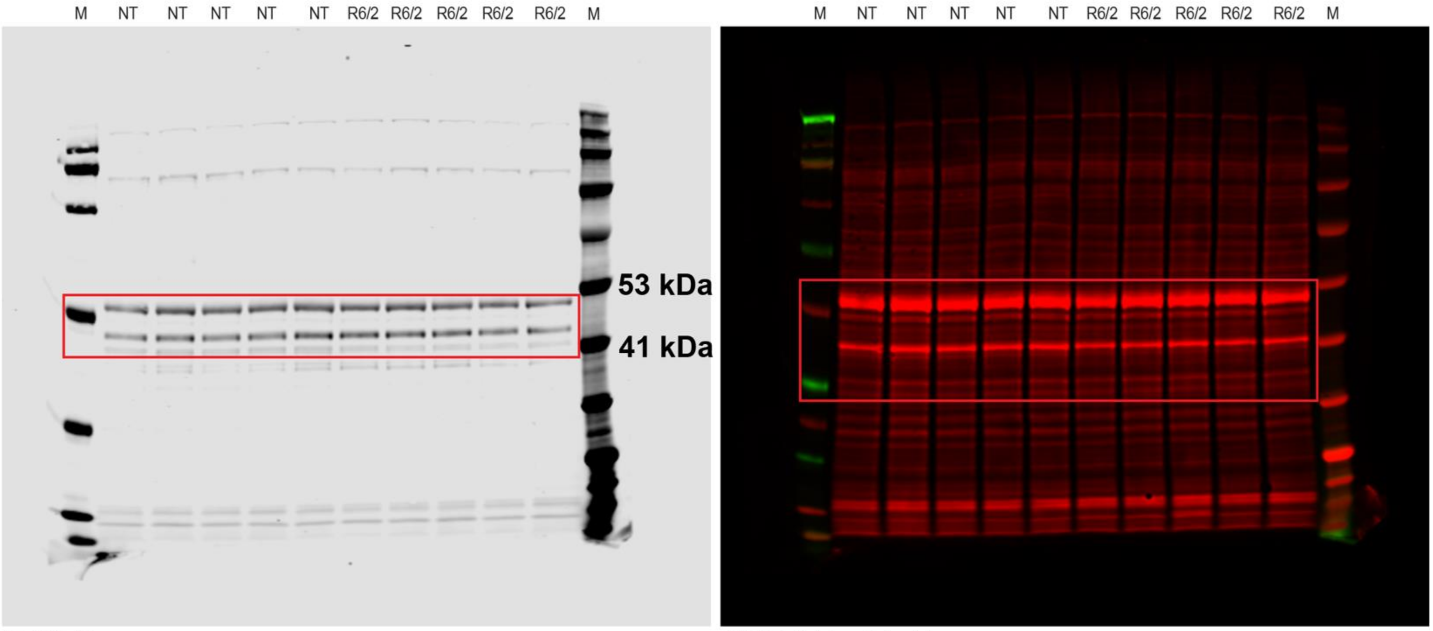

IB: TDP-43

REVERT total protein stain

B.

Uncropped blots for Supplementary Figure 5D

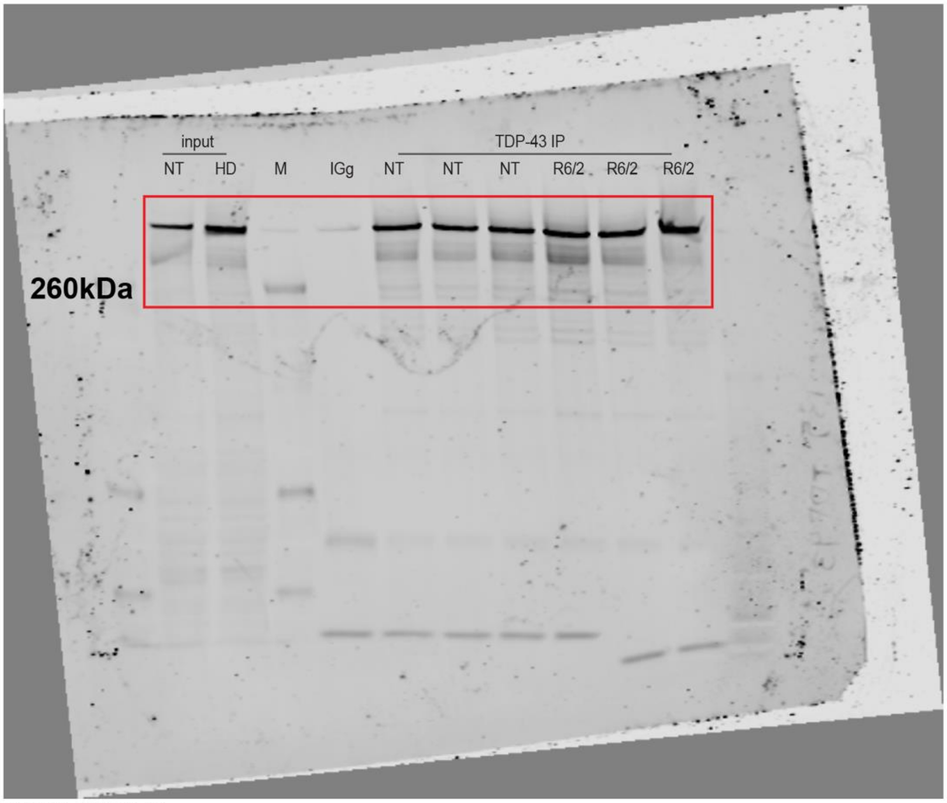

IB: HTT, IP: TDP-43

Supplementary Figure 9. Uncropped Western Blots.

Blots are scanned on Li-COR western machine, (A) Uncropped western blot for Supplementary Figure 5C, left blot shows western blot, right blot shows Revert total protein stain. Red box indicates areas cropped for figure. (B) Uncropped Co-IP western blot for Supplementary Figure 5D. Red box indicates areas cropped for figure.

Supplementary Figure 10

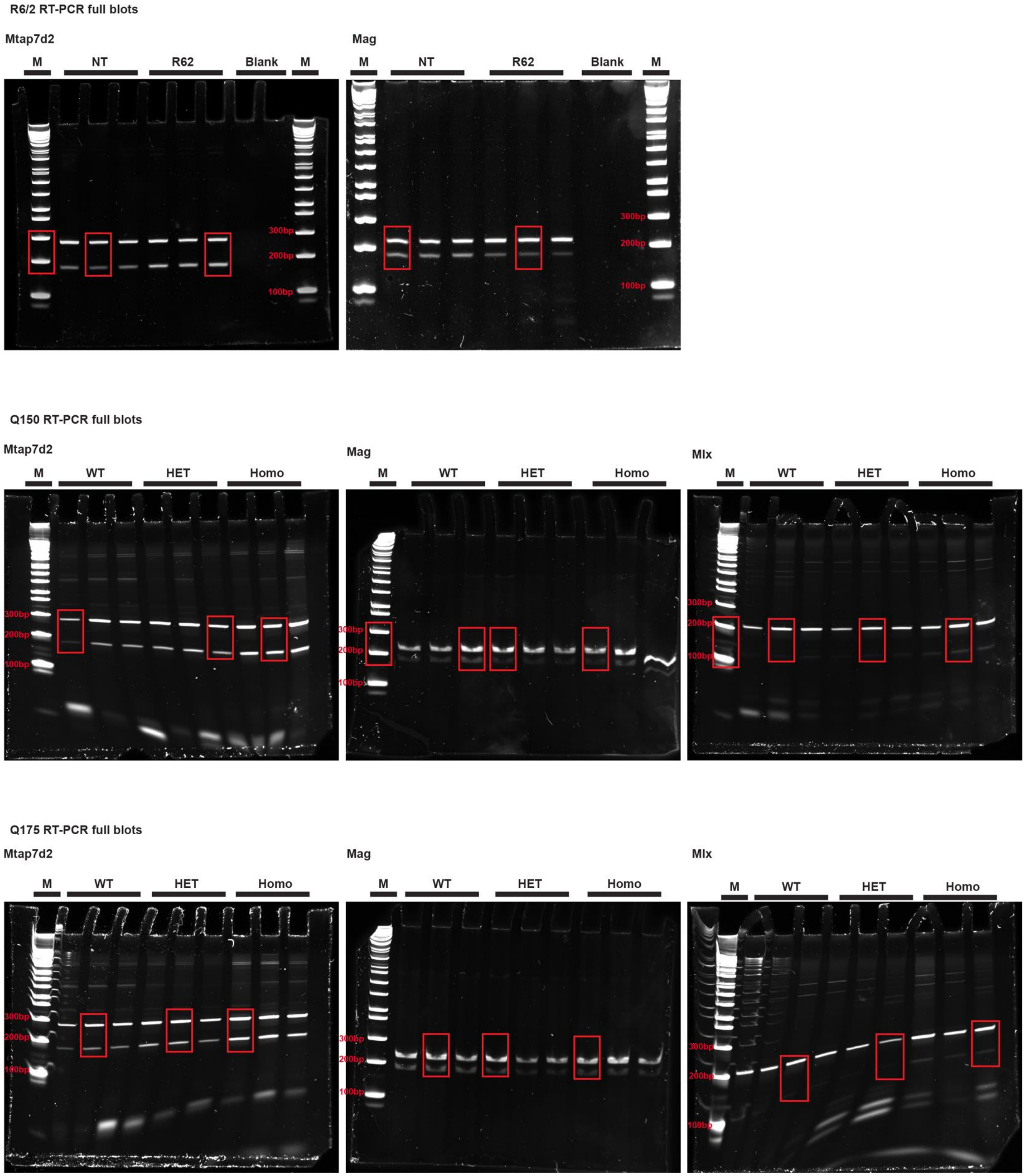

Supplementary Figure 10. Uncropped RT-PCR DNA gel.
Uncropped RT-PCR DNA gel for Supplementary Figure 2B. Red box indicate section used for figure.

Supplementary Figure 11

Uncropped blots for Figure 3E

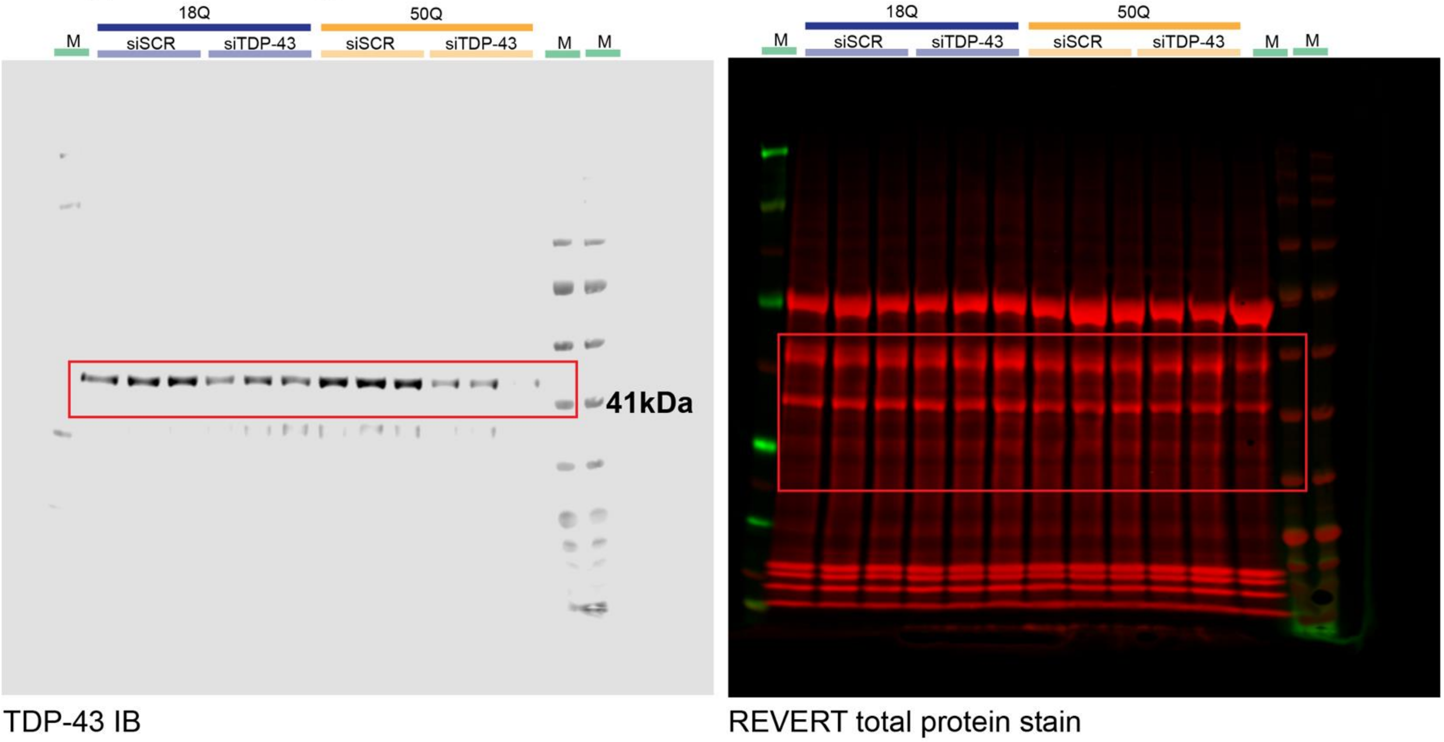

Supplementary Figure 11. Uncropped blots for Figure 3E.
Uncropped blots for figures 3E, red box indicate cropped area used for figure.

Supplementary Data Legends:

Supplementary Data 1:
Column A, List of RNA-binding proteins that are enriched in the HDinHD proteome dataset [10]. Column C, List of RNA-binding proteins predicted from SONAR [11].

Supplementary Data 2:
Differential gene expression data from DESeq2 [4]. N=10(5 males, 5 females) per genotype were used for analysis. Statistical p-values listed are performed within the DESeq2 package. Left side shows Cortex data, and right side shows Striatum data. Filtered for events with padj<0.05.

Supplementary Data 3:
List of CHDI's 266 Striatal genes that are dysregulated in HD mouse models [5]. Column C shows direction of change relative to HD condition.

Supplementary Data 4:
List of significant (FDR <0.05) Skipped Exons (SE) as annotated by rMATS [6]. Cortex, rows 3 – 3524. Striatum, rows 3529-7689. Human, (Al-Dalahmah et al [12], Human SE), rows 7691-12372. Human, (Labadorf et al [13], Human SE, Human SE), rows 12377-17821. P-values are determined and output by rMATS.

Supplementary Data 5:
Output from MAJIQ and Leafcutter. Dataset was filtered for probability_changing>.90 for MAJIQ and deltapsi>.10 or -.10 MAJIQ Cortex, rows 3-203. MAJIQ Striatum, rows 207-443. Leafcutter Cortex, rows 447-610. Leafcutter Striatum, rows 614-857.

Supplementary Data 6:
Differential gene expression data from DESeq2 [4]. DESeq2 output from HTT(18Q) vs mHTT(50Q) Day 37 MSNs. n=3 per genotype were used for analysis. Statistical p-values listed are performed within the DESeq2 package. Filtered for events with padj<0.05.

Supplementary Data 7:
Differential gene expression data from DESeq2 [4]. DESeq2 output DESeq2 output from HTT(18Q) non-targeting siRNA vs TDP-43 knockdown siRNA at Day 37 MSNs. n=3 per genotype were used for analysis. Statistical p-values listed are performed within the DESeq2 package. Filtered for events with padj<0.05.

Supplementary Data 8:
List of differentially expressed genes that overlapped between TDP-43 knockdown and mHTT-dependent (p-value < 0.05) as determined by the hypergenometric test. Column A are genes overlapping with upregulated genes in the mHTT condition and column B are overlap with downregulated genes in the mHTT condition.

Supplementary Data 9:
Summary of human post-mortem brain tissues used in this study. M=male, F=female. HD-X, with X indicating Vonsattel grading. PMD = Post-mortem delay.

Supplementary Data 10:
List of antibodies used in this study.

Supplementary Data 11:
List of primer pairs used for RASL-seq

Supplementary Data 12:
RASL-seq raw data for R6/2 (starting at row 1), Q150 (starting at row 1748), and Q175 (starting at row 3538).

References:

1. Harris, M.A., et al., *The Gene Ontology (GO) database and informatics resource.* Nucleic Acids Res, 2004. **32**(Database issue): p. D258-61.
2. Eden, E., et al., *GOrilla: A tool for discovery and visualization of enriched GO terms in ranked gene lists.* BMC Bioinformatics, 2009. **10**: p. 1-7.
3. Liao, Y., G.K. Smyth, and W. Shi, *featureCounts: an efficient general purpose program for assigning sequence reads to genomic features.* Bioinformatics, 2014. **30**(7): p. 923-30.
4. Love, M.I., W. Huber, and S. Anders, *Moderated estimation of fold change and dispersion for RNA-seq data with DESeq2.* Genome Biology, 2014. **15**(12): p. 1-21.
5. Obenauer, J.C., et al., *Expression analysis of Huntington disease mouse models reveals robust striatum disease signatures.* BioRxiv, 2022.
6. Shen, S., et al., *rMATS: Robust and flexible detection of differential alternative splicing from replicate RNA-Seq data.* Proceedings of the National Academy of Sciences of the United States of America, 2014. **111**(51): p. E5593-E5601.
7. Li, H., J. Qiu, and X.D. Fu, *RASL-seq for Massively Parallel and Quantitative Analysis of Gene Expression.* Current Protocols in Molecular Biology, 2012. **1**(SUPPL.98).
8. Šušnjar, U., et al., *Cell environment shapes TDP-43 function with implications in neuronal and muscle disease.* Communications Biology, 2022. **5**(1).
9. Olarerin-George, A.O. and S.R. Jaffrey, *MetaPlotR: A Perl/R pipeline for plotting metagenes of nucleotide modifications and other transcriptomic sites.* Bioinformatics, 2017. **33**(10): p. 1563-1564.
10. Aaronson, J., et al., *HDinHD: A Rich Data Portal for Huntington's Disease Research.* Journal of Huntington's Disease, 2021. **10**(3): p. 405-412.
11. Brannan, K.W., et al., *SONAR Discovers RNA-Binding Proteins from Analysis of Large-Scale Protein-Protein Interactomes.* Molecular Cell, 2016. **64**(2): p. 282-293.
12. Al-Dalahmah, O., et al., *Single-nucleus RNA-seq identifies Huntington disease astrocyte states.* Acta Neuropathologica Communications, 2020. **8**(1): p. 19.
13. Labadorf, A., et al., *RNA sequence analysis of human huntington disease brain reveals an extensive increase in inflammatory and developmental gene expression.* PLoS ONE, 2015. **10**(12).
