# Supplementary material for: Aberrant splicing in Huntington’s disease accompanies disrupted TDP-43 activity and altered m6A RNA modification
Source: Nat Neurosci. 2025 Jan 6;28(2):280–92. doi: 10.1038/s41593-024-01850-w (PMC11802453; doi:10.1038/s41593-024-01850-w)
Supplement: Supplementary file 2 — Reporting Summary [file 41593_2024_1850_MOESM2_ESM.pdf]

nature portfolio

Corresponding author(s): Leslie M Thompson

Last updated by author(s): Oct 1, 2024

Reporting Summary

Nature Portfolio wishes to improve the reproducibility of the work that we publish. This form provides structure for consistency and transparency in reporting. For further information on Nature Portfolio policies, see our Editorial Policies and the Editorial Policy Checklist.

Statistics

For all statistical analyses, confirm that the following items are present in the figure legend, table legend, main text, or Methods section.

n/a	Confirmed	
☐	☒	The exact sample size (*n*) for each experimental group/condition, given as a discrete number and unit of measurement
☐	☒	A statement on whether measurements were taken from distinct samples or whether the same sample was measured repeatedly
☐	☒	The statistical test(s) used AND whether they are one- or two-sided<br>*Only common tests should be described solely by name; describe more complex techniques in the Methods section.*
☐	☒	A description of all covariates tested
☐	☒	A description of any assumptions or corrections, such as tests of normality and adjustment for multiple comparisons
☐	☒	A full description of the statistical parameters including central tendency (e.g. means) or other basic estimates (e.g. regression coefficient) AND variation (e.g. standard deviation) or associated estimates of uncertainty (e.g. confidence intervals)
☐	☒	For null hypothesis testing, the test statistic (e.g. *F*, *t*, *r*) with confidence intervals, effect sizes, degrees of freedom and *P* value noted<br>*Give P values as exact values whenever suitable.*
☒	☐	For Bayesian analysis, information on the choice of priors and Markov chain Monte Carlo settings
☒	☐	For hierarchical and complex designs, identification of the appropriate level for tests and full reporting of outcomes
☒	☐	Estimates of effect sizes (e.g. Cohen's *d*, Pearson's *r*), indicating how they were calculated

Our web collection on statistics for biologists contains articles on many of the points above.

Software and code

Policy information about availability of computer code

Data collection	Immunobloting was performed on LiCOR Odyssey CLx with Image Studio (version 5.2) & Empiria (version 2.0), Immunofluorescence (IF) imaging was collected on the Olympus Fluoview FV3000 and the Zeiss LSM 900 Airyscan 2. image postprocessing - IMARIS (version 10), qPCRs were performed on QuantaStudio 5 (Thermo), High-throughput sequencing was performed with the NovaSeq6000, HiSeq2000, Sequel II platform. LC-MS, quadrupole-orbitrap mass spectrometer coupled to hydrophilic interaction chromatography (HILIC) via electrospray ionization.
Data analysis	Statistical analysis Prism (graphpad version 10.0.1). Cellprofiler (v4.2.6.). RNA-seq: Reference genome (Human, Homo_sapiens.GRCh38.104.gtf, Homo_sapiens.GRCh38.dna.primary_assembly.fa) (mouse, gencode.vM25.annotation.gtf, GRCm38.p6.genome.fa) (FASTQC (v0.11.7), DESeq2 (version v1.42.0), STAR aligner (v2.7.0), edgeR (v4.0.16) , de novo Motif finding - HOMER (version 4.11.1). Pacbio ISO-seq: SMRT Link v9.0, ccs 4.2.0 , minimap2 (2.17-r941), TranscriptClean (v2.0.2), TALON (v5.0). eCLIP analysis - CLIPPER (https://github.com/YeoLab/clipper), , MetaplotR (https://github.com/olarerin/metaPlotR), DESJ-detetion (version 2.0.5), Deeptools (version 3.5.1), ggpplot2 (version 3.4.1). CE analysis: MAJIQ (v2.4.dev3+g85d0781), Leafcutter (v0.2.9), rMATS (v4.1.1). LC-MS, El-MAVEN software (version 0.12.0).

For manuscripts utilizing custom algorithms or software that are central to the research but not yet described in published literature, software must be made available to editors and reviewers. We strongly encourage code deposition in a community repository (e.g. GitHub). See the Nature Portfolio guidelines for submitting code & software for further information.

Data

Policy information about availability of data

All manuscripts must include a data availability statement. This statement should provide the following information, where applicable:

- Accession codes, unique identifiers, or web links for publicly available datasets
- A description of any restrictions on data availability
- For clinical datasets or third party data, please ensure that the statement adheres to our policy

Public dataset used in this study were: TDP-43 iCLIP & ASO KD (GEO: GSE27394), Al-Dalahmah et al., 2020 - (requested from author), Labadorf et al., 2015 (GEO: GSE64810), and Šušnjar et al., 2022 (GEO: GSE171714). All sequencing raw data generated in this study are deposited in GEO, with the accession: GSE278354, GSE278893, GSE279460. All data are available in the main text or the supplementary materials, all other data can be made available with reasonable requests.

Research involving human participants, their data, or biological material

Policy information about studies with human participants or human data. See also policy information about sex, gender (identity/presentation), and sexual orientation and race, ethnicity and racism.

Reporting on sex and gender	The focus of this study was to look at the CAG length of Huntingtin. Due to limiting case samples, our analysis could not independently consider sex and gender. Both sexes were used in this study.
Reporting on race, ethnicity, or other socially relevant groupings	N/A
Population characteristics	Post-mortem human brains were obtained from the New Zealand Brain bank (Control (mean PMD 5.52): 3 males, 2 females, mean age 80 || HD (mean PMD 6.14): 3 males, 2 females, mean age 63) and The Netherlands Brain Bank ((Control (mean PMD 20.37): 5 males, 3 females, mean age 56 || HD (mean PMD 16.88): 7 males, 5 females, mean age 63). The brain samples and/or bio samples were obtained from The Netherlands Brain Bank, Netherlands Institute for Neuroscience, Amsterdam (open access: www.brainbank.nl).
Recruitment	All Material has been collected from donors for or from whom a written informed consent for a brain autopsy and the use of the material and clinical information for research purposes had been obtained by the NBB. Informed consent was obtained in writing from all participant families as required by the Health and Disability Ethics Committee by NZBB.
Ethics oversight	The Netherlands Brain Bank, The New Zealand Brain bank. Approved by Health and Disability Ethics Committee (Ethics number: 14/NTA/208/AM02), Ministry of Health.

Note that full information on the approval of the study protocol must also be provided in the manuscript.

Field-specific reporting

Please select the one below that is the best fit for your research. If you are not sure, read the appropriate sections before making your selection.

☒ Life sciences ☐ Behavioural & social sciences ☐ Ecological, evolutionary & environmental sciences

For a reference copy of the document with all sections, see nature.com/documents/nr-reporting-summary-flat.pdf

Life sciences study design

All studies must disclose on these points even when the disclosure is negative.

Sample size	n=10 per genotype (5 per male/female) were used for mouse sequencing studies, and were determined based on previously published literature. The most amount of samples were used per conditions as feasible. No statistical methods were used to pre-determine sample sizes, however sample size selections were made to be comparable to previous published studies. IF studies sample selection were based on sample availability, and conclusions drawn were appropriate for the sample size.
Data exclusions	No data was excluded
Replication	multiple RNA sequencing techniques were performed in multiple labs were used to validate findings. Experiments were repeated at least 3 times with similar results to those presented in the manuscript.
Randomization	only genotype condition were considered in this study, thus randomization were not used. for TDP-43 KD, random samples were chosen to receive control scramble siRNAs or TDP-43 siRNA.
Blinding	Researchers were blinded to conditions during analysis. Researchers could not be blinded during group allocations due to the distinct behavioral characteristics of the animals used in this study.

Reporting for specific materials, systems and methods

We require information from authors about some types of materials, experimental systems and methods used in many studies. Here, indicate whether each material, system or method listed is relevant to your study. If you are not sure if a list item applies to your research, read the appropriate section before selecting a response.

Materials & experimental systems

n/a	Involved in the study
☐	☒ Antibodies
☐	☒ Eukaryotic cell lines
☒	☐ Palaeontology and archaeology
☐	☒ Animals and other organisms
☒	☐ Clinical data
☒	☐ Dual use research of concern
☒	☐ Plants

Methods

n/a	Involved in the study
☒	☐ ChIP-seq
☒	☐ Flow cytometry
☒	☐ MRI-based neuroimaging

Antibodies

Antibodies used	HTT (EM48) - Millipore Sigma Cat#MAB5374 // HTT (MW8) - DSHB Cat#AB_528297 // HTT (5492) - Millipore Sigma Cat#MAB5492 // HTT (D7F7) - Cell Signaling Cat#5656 // HTT (EPR5526) - Abcam Cat#ab109115 // TDP-43 - Proteintech Cat#12892-1-AP // TDP-43 - Proteintech Cat#10782-2-AP // TDP-43 - Abcam Cat#[3H8] ab104223 // TDP-43 - Bethyl Cat#A303-223A // Phospho-TDP-43 - Gift from Dr. Leonard Petrucelli Cat#RB3655 // Phospho-TDP-43 - Biolegend Cat#829901 // MAP2 - SYSY Cat#188004 // m6A Abcam Cat# ab92821 //m6A - Sy Sy Cat# 203-003 // METTL3 - Abcam Cat#[EPR18810] ab195352 // METTL14 - Millipore Sigma Cat#HPA038002 // FTO - Abcam Cat#[5-2H10] ab92821 // ALKBH5 - Millipore Sigma Cat#HPA007196 // RBM15 - Proteintech Cat#10587-1-AP // WTAP Santa Cruz Biotechnology Cat#sc-374280. Additional information can be found on supplemental table S9.
Validation	All primary antibodies were commercially purchased and chosen based on previous published studies in comparable sample types. Antibodies for IB were validated using LiCOR standard validation protocol for linear range and dilutions. Antibodies were selected with knockout or over-expression validation when possible, unless it is a standard antibody accepted in the field. The validation information for each antibody is available from the manufacturers' websites.

Eukaryotic cell lines

Policy information about cell lines and Sex and Gender in Research

Cell line source(s)	Parental cell line was (CS83iCTR33-n1 (RRID:CVCL_IW28)) obtained from the Cedar Sinai iPSC core. All the cell lines and protocols in the present study were carried out in accordance with the guidelines approved by institutional review boards at the UCI and JHU. Studies were performed under the auspices of the UCI Institutional Review Board (IRB) approved protocol UCI IRB #2008-6556.Maintenance and differentiation of iPSCs were performed at UCI in accordance with the protocol at UCI hSCRO #118. Appropriate informed consents were obtained from all the donors.
Authentication	CRISPR edited isogenic iPSC lines harboring 18Q and 50Q CAG repeats in HTT exon 1 was sent for sequencing to determine CAG repeat size. lines were authenticated for pluripotency and differentiation abilities. G-band karyotyping/aCgH array was performed to ensure that iPSCs maintained normal karyotypes (46XX or 46XY where appropriate).
Mycoplasma contamination	Cells are routinely tested for Mycoplasma and are considered negative.
Commonly misidentified lines (See ICLAC register)	There are no commonly misidentified lines used in this study.

Animals and other research organisms

Policy information about studies involving animals; ARRIVE guidelines recommended for reporting animal research, and Sex and Gender in Research

Laboratory animals	Mice strain purchased from Jackson Laboratories: B6CBA-Tg(HDexon1)62Gpb/3J, B6.129P2-Htttm2Detl/150J, B6J.129S1-Htttm1Mfc/190ChdiJ, and C57BL/6J. Mice were received at ~5 weeks of age, and were used at ages 8 and 12 weeks for the study were . Animals were housed at controlled temperature: 70F and 50% humidity.
Wild animals	This study did not involve wild animals.
Reporting on sex	Initial transcriptome analysis were performed with males and females separately, with minimal differences. Thus subsequent analysis were performed with both males and female animals, and accounting for sex differences.
Field-collected samples	This study did not include samples collected from the field.

Ethics oversight

All experimental procedures were in accordance with the Guide for the Care and Use of Laboratory Animals of the NIH and animal protocols were approved by Institutional Animal Care and Use Committees at the University of California Irvine (UCI), an AAALAC accredited institution - PROTOCOL # AUP-21-087. RASL-seq, All procedures were conducted in accordance with the guidelines of the University of California San Diego Institutional Animal Care and Use Committee (#S0022)

Note that full information on the approval of the study protocol must also be provided in the manuscript.
